# New copper carboxyl­ate pyrene dimers: synthesis, crystal structure, Hirshfeld surface analysis and electrochemical characterization

**DOI:** 10.1107/S2056989023010277

**Published:** 2024-01-01

**Authors:** Vianca C. Nogué-Guzmán, Alejandro Burgos-Suazo, Javier O. Rivera-Reyes, Vasti P. Montes Quiñones, Paola C. Ramis-Aybar, Adriana C. Burgos-Jiménez, Karilys González-Nieves, Dalice M. Piñero-Cruz

**Affiliations:** a Department of Natural Sciences, University of Puerto Rico, Carolina Campus, Carolina, 00984-4800, Puerto Rico; b Department of Chemistry, University of Puerto Rico, Rio Piedras Campus, San Juan, 00927, Puerto Rico; c University of Puerto Rico’s Molecular Sciences Research Center, San Juan, 00926, Puerto Rico; Universidad de la Repüblica, Uruguay

**Keywords:** dicopper, carboxyl­ate, pyrene, π–π stacking, crystal structure

## Abstract

Two new copper dimers, namely, bis­(dimethyl sulfoxide)­tetra­kis­(μ-pyrene-1-carboxyl­ato)dicopper(*Cu*—*Cu*), [Cu_2_(C_17_H_9_O_2_)_4_(C_2_H_6_OS)_2_] or [Cu_2_(pyr-COO^−^)_4_(DMSO)_2_] (**1**), and bis­(di­methyl­formamide)­tetra­kis­(μ-pyrene-1-carboxyl­ato)dicopper(*Cu*—*Cu*), [Cu_2_(C_17_H_9_O_2_)_4_(C_3_H_7_NO)_2_] or [Cu_2_(pyr-COO^−^)_4_(DMF)_2_] (**2**) (pyr = pyrene), were synthesized from the reaction of pyrene-1-carb­oxy­lic acid, copper(II) nitrate and tri­ethyl­amine from solvents DMSO and DMF, respectively. While **1** crystallized in the space group *P*  , the crystal structure of **2** is in space group *P*2_1_/*n*. The Cu atoms have octa­hedral geometries, with four oxygen atoms from carboxyl­ate pyrene ligands occupying the equatorial positions, a solvent mol­ecule coordinating at one of the axial positions, and a Cu⋯Cu contact in the opposite position. The packing in the crystal structures exhibits π–π stacking inter­actions and short contacts through the solvent mol­ecules. The Hirshfeld surfaces and two-dimensional fingerprint plots were generated for both compounds to better understand the inter­molecular inter­actions and the contribution of heteroatoms from the solvent ligands to the crystal packing. In addition, a Cu^2+^/Cu^1+^ quasi-reversible redox process was identified for compound **2** using cyclic voltammetry that accounts for a diffusion-controlled electron-donation process to the Cu dimer.

## Abstract 2

Two new copper dimers, [Cu_2_(pyr-COO–)_4_(DMSO)_2_] (**1**) and [Cu_2_(pyr-COO–)_4_(DMF)_2_] (**2**) (pyr = pyrene) were synthesized from the reaction of pyrene-1-carb­oxy­lic acid, copper(II) nitrate and tri­ethyl­amine from solvents DMSO and DMF, respectively. Electrochemical characterization and Hirshfeld surface analysis was carried out.

## 1. Chemical context

Copper(II) carboxyl­ate complexes with paddle-like structure have been proposed in solar energy conversion and storage, redox mediators, magnetism, dyes and in catalysis, among other applications (Benesperi *et al.* 2020 ▸; Kozlevčar *et al.*, 2004 ▸; Rajakannu *et al.*, 2019 ▸; Murugavel *et al.*, 2000 ▸; Rao *et al.*, 2004 ▸; Boulsourani *et al.*, 2017 ▸; Baldomá *et al.*, 2006 ▸; Seo *et al.*, 2000 ▸). The unique characteristics of copper(II) carboxyl­ate complexes of general formula [Cu_2_(RCOO^−^)_4_(*L*)_2_] are based on their easy synthesis, the relative abundance of the starting materials, their stability, and their low toxicity, which enables a vast number of research directions to be performed from such materials. The structural features of these compounds are related to the coordinating aspect of the ligands: the two possible coordination sites through the carboxyl­ate oxygen atoms result in various modes of coordination, such as monodentate, bidentate and bridging, offering a variability of polynuclear metal complexes (Rajakannu *et al.*, 2019 ▸; Murugavel *et al.*, 2000 ▸; Rao *et al.*, 2004 ▸), and a degree of stability for dinuclear and trinuclear complexes. Additionally, the carboxyl­ate group could participate in hydrogen bonds, leading to a supra­molecular network (Aakeröy *et al.*, 2006 ▸). Moreover, dinuclear copper(II) carboxyl­ate complexes may have switchable electronic properties such as inter­metal magnetic exchange and electron transfer (Vishnoi *et al.*, 2017 ▸). The electrochemical properties of copper(II) carboxyl­ate complexes are reported to be highly influenced by the redox-active nature of copper(II/I) and subjected to potential changes due to the presence of substituents in the carboxyl­ate ligands (Wang *et al.*, 2013 ▸), thereby influencing the stability of its oxidation state (Modec *et al.*, 2020 ▸). 
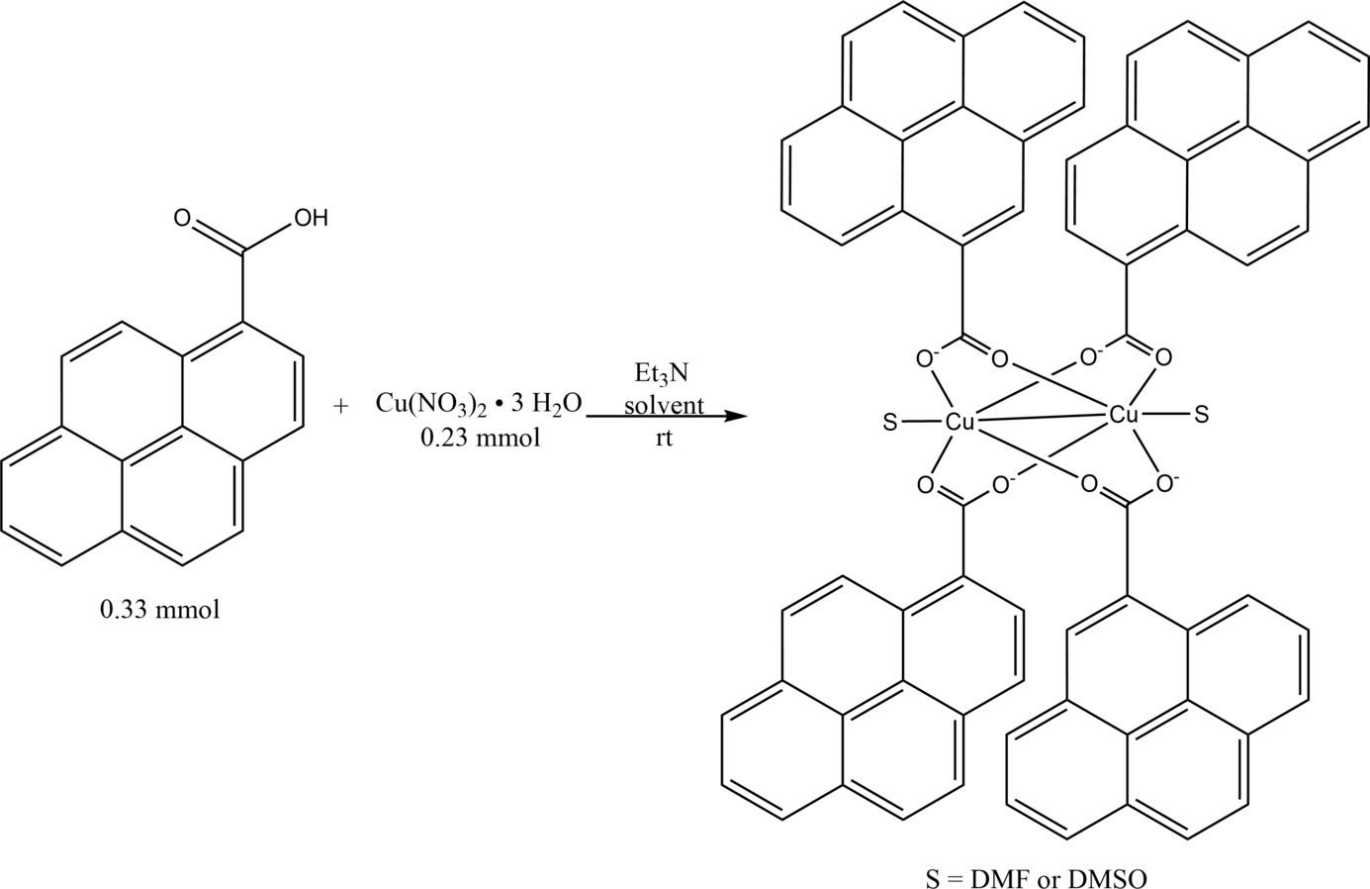


In this work we report the structure of two new copper(II) carboxyl­ate complexes from pyrene-1-carb­oxy­lic acid. The structure of pyrene is based on four fused benzene rings, thus it belongs to the group of polycyclic aromatic hydro­carbons (PAH) that have been well studied since their remarkable fluorescence and phospho­rescence properties were noted (Haldar *et al.*, 2020 ▸). Carb­oxy­lic acid from 1-pyrene has been proposed in supercapacitor devices by functionalization of graphene and it is also used to design and synthesize lumin­escent metal–organic complexes for sensing applications.

In addition, pyrene ligands have been used as an organic linker or as building blocks for the design of new classes of metal–organic frameworks (MOFs). The functionalization of pyrene with phospho­nates, sulfonates, and carboxyl­ates allows metal coordination to yield MOF structures exhibiting new photophysical and photochemical properties. MOF structures with pyrene ligands result in promising optical properties such as luminescence sensing, photocatalysis, electrochemistry, adsorption and separation applications, and biomedical applications (Kinik *et al.*, 2021 ▸). Derivatives of carboxyl­ate pyrene ligands have been studied because of their extraordinary photophysical properties, chemical stability, π–π stacking inter­actions, and high-charge-delocalized systems (Guan *et al.*, 2019 ▸). The planar π-conjugated surface of pyrene and its mol­ecular rearrangement is favorable for the detection of guest mol­ecules in mol­ecular tweezer hosts, for example with platinum, ruthenium, and copper complexes. Another application of pyrene can be found in the functionalization of carbon nanotubes (CNTs) as a result of its π–π inter­actions with polycyclic aromatic mol­ecules (Zhao & Stoddart, 2009 ▸).

Here, we report the novel synthesis, characterization, and crystal structure of two copper dimers with tetra­carboxyl­ate pyrene and two solvent mol­ecules in axial positions, [Cu_2_(pyr-COO^−^)_4_(DMSO)_2_] (**1**) and [Cu_2_(pyr-COO^−^)_4_(DMF)_2_] (**2**). Structural characterization from single-crystal X-ray diffraction experiments show crystallization under two crystal systems, which translates into different extended contacts, such as π–π stacking inter­actions, among others. In terms of the chemistry of these copper structures, they are very promising because the axial positions can be substituted by bridging ligands, which can form coordination polymers such as the 1D, 2D, and 3D polymeric architectures that have been proposed in mol­ecular sensing, gas storage, and separation (Karmakar *et al.*, 2021 ▸). Hirshfeld surface analysis was undertaken to show the contributions from inter­molecular inter­actions in the crystal-packing array. The pyrene rings participate in π–π inter­actions, yet some rings have weaker inter­actions based on their position in the crystal structure. DMSO (**1**) and DMF (**2**) axial ligands, play a crucial role in the crystal packing by participating in inter­actions with the rest of the mol­ecule. In addition, Hirshfeld surface analysis showed that compound **2** has shorter distances for most inter­actions. Electrochemical characterization of compound **2** was performed by cyclic voltammetry at varying scan rates (50–2000 mV s^−1^), revealing a diffusion-controlled Cu^2+^/Cu^1+^ quasi-reversible process that may involve an electron reduction at an *E*_1/2_ potential around −0.52 V *vs F*_c_/*F*_c_^+^ (Iqbal *et al.*, 2013 ▸; Bonomo *et al.*, 2000 ▸).

## 2. Structural commentary

**Figure 1:**
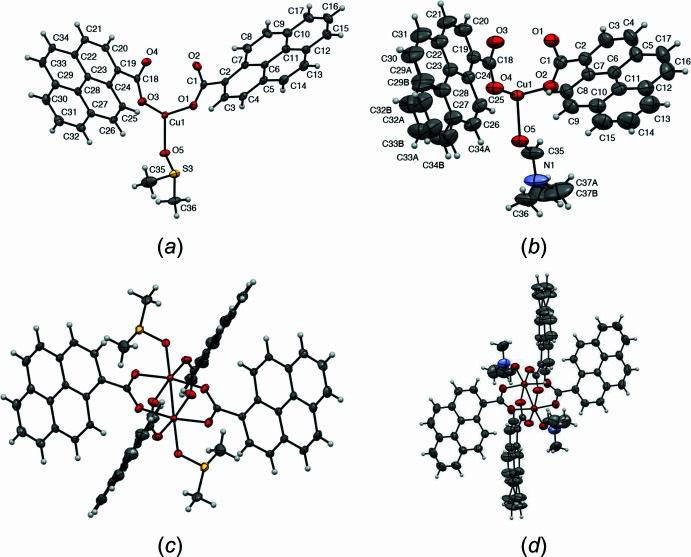
Asymmetric units of Cu complexes **1** (*a*) and **2** (*b*) with labels for non-C/H atoms and ellipsoids at the 50% probability level. (*c*) and (*d*) views of the complete mol­ecules from the *b* axis.

The crystal structures of complexes [Cu_2_(pyr-COO^−^)_4_(DMSO)_2_] (**1**), space group *P* 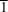 , and [Cu_2_(pyr-COO^−^)_4_(DMF)_2_], space group *P*2_1_/*n* (**2**), are presented in Fig. 1 ▸. The copper atoms have octa­hedral geometries with four oxygen atoms from the pyrene-1-carboxyl­ate ligand at equatorial positions, one axial ligand from the solvent mol­ecule and the remaining axial coordination occupied by a metal–metal copper contact. The asymmetric unit contains half the mol­ecule in both structures. The Cu⋯Cu contact distance in **1** is 2.5934 (3) Å in comparison with the structure of **2** for which it is 2.6295 (5) Å. Likewise, the Cu—O5 bond distance in the axial position is shorter in **1** than in **2**, with values of 2.1441 (12) and 2.1769 (13) Å, respectively. The difference in the elongation of these bond distances could be the result of the influence of the axial ligand (DMSO *vs* DMF) with stronger π-back-bonding character, thus better binding (Deacon & Phillips, 1980 ▸). The Cu—O bonds in equatorial positions are shorter than those in axial positions in both structures, with distances ranging from 1.9530 (13) to 1.9593 (13) Å, which may be indicative of Jahn–Teller effects on Cu^II^ centers. All the other structural features in the two Cu dimers do not change significantly. Structural disorder of four carbon atoms from the pyrene (C29–C32—C33—C34) unit is observed in complex **2** as well as in one of the carbon atoms from the DMF mol­ecule, precisely on C37, for which atoms had to be modeled in two parts.

## 3. Supra­molecular features

**Figure 2:**
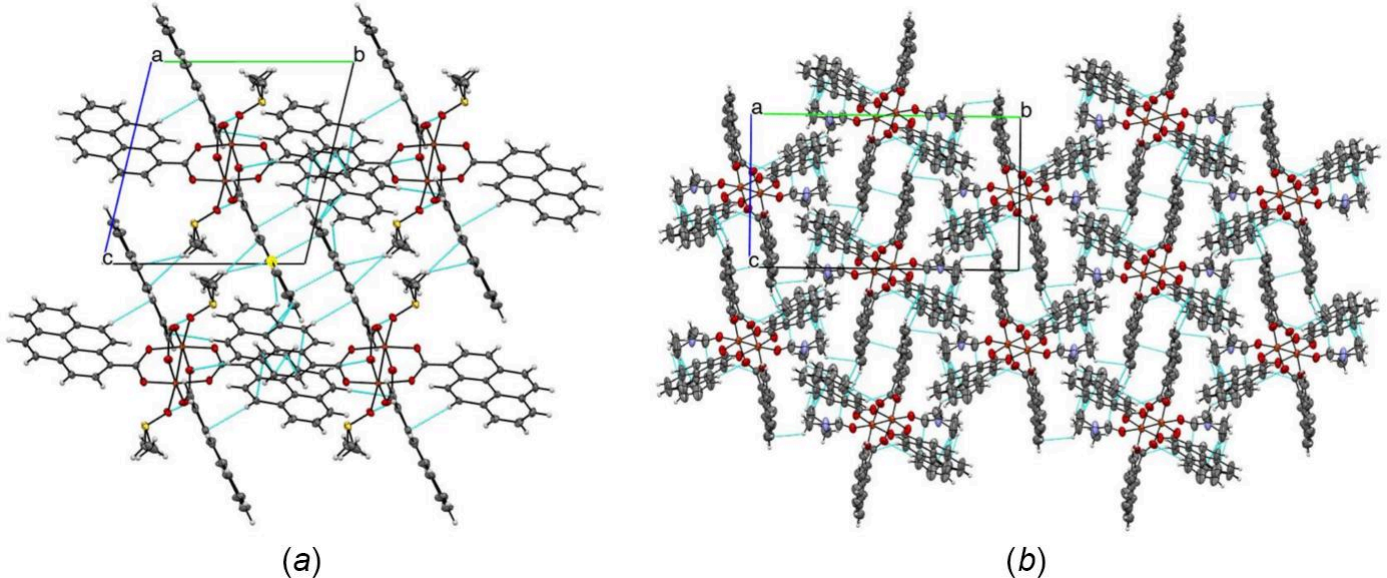
Crystal packing of Cu complexes **1** and **2** along the *a* axis with ellipsoids at the 50% probability level.

Long-range inter­actions for **1** and **2** are different in terms of their π–π stacking, as well as the axial hydrogen inter­actions with π rings. In the case of complex **1**, the most important π–π inter­actions is observed for C22⋯C16 at 3.393 (3) Å. C—H to π-ring inter­actions are observed between C28⋯H15 and C27⋯H15 at 2.87 and 2.90 Å, respectively, and the solvent oxygen inter­action π-ring end hydrogen is observed through O5⋯H4 at a distance of 2.56 Å. In complex **2** however, π–π inter­actions are present from C4⋯C4 of neighboring rings with a distance 3.178 (4) Å; other inter­actions are attributed to C—H end to π-ring for C16⋯H37*B*, C19⋯H16, and C24⋯H16 with distances of 2.87, 2.85, and 2.70 Å, respectively. The packing for **1** and **2** is shown in shown in Fig. 2 ▸.

## 4. Electrochemical measurements

**Figure 3:**
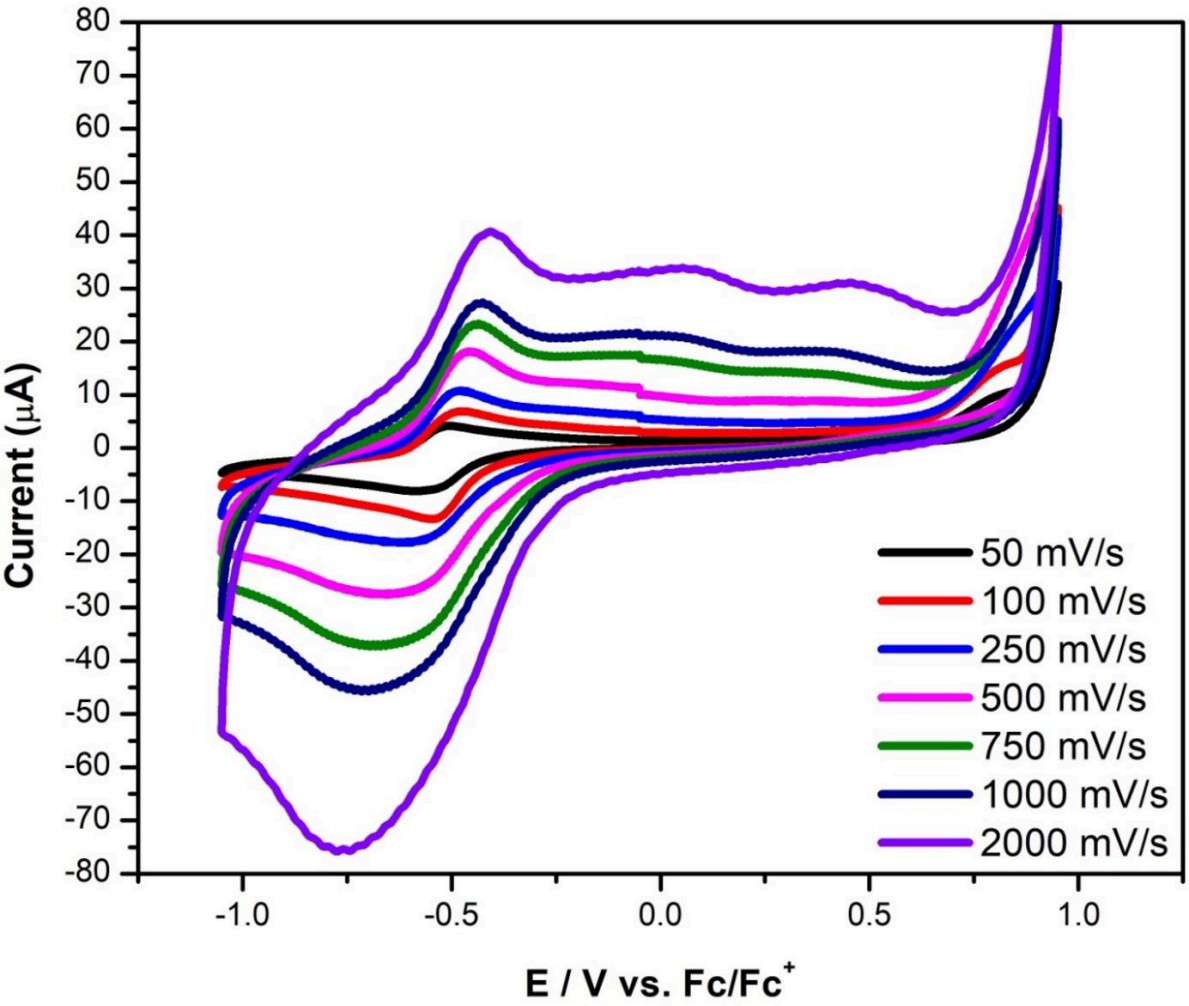
Cyclic voltammograms of 1 m*M* of compound **2** at 50–2000 mV s^−1^. Cyclic voltammograms were obtained in a 0.1 *M* TBAPF_6_ in DMF with a glassy carbon working electrode, a graphite rod counter-electrode, and 0.01 *M* AgNO_3_ silver wire as the pseudo-reference electrode corrected with ferrocene.

**Figure 4:**
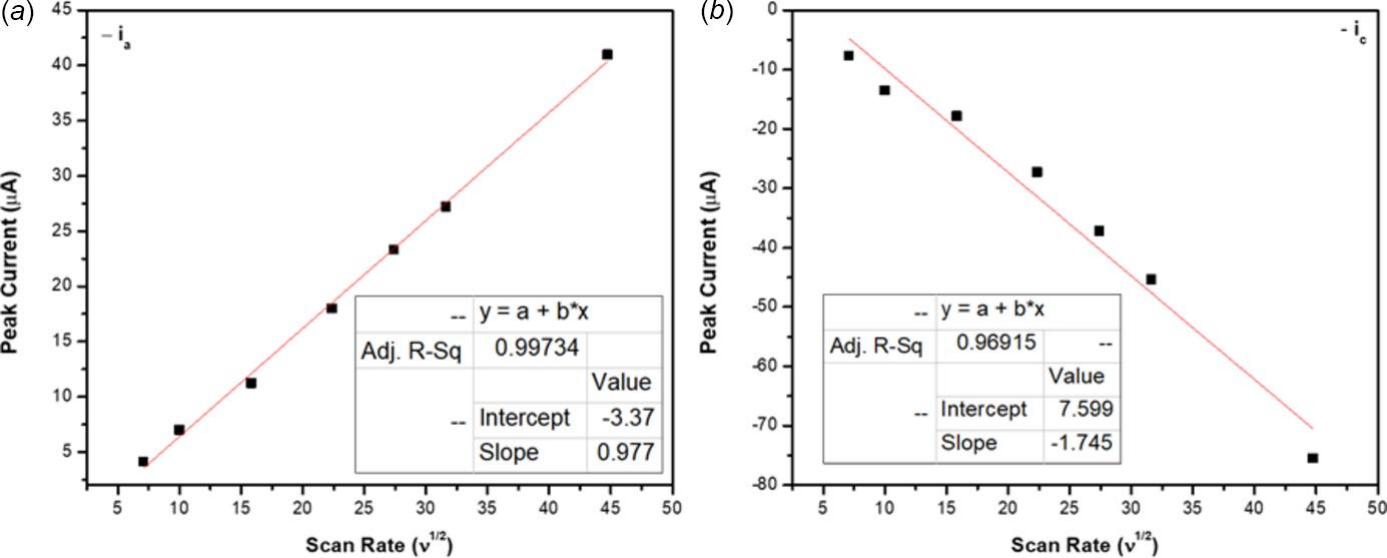
Square root of scan rate *versus* peak current plot (*a*) anodic peak and (*b*) cathodic peak of compound **2**.

Electrochemical properties were measured in DMF for complex **2**; complex **1** was not soluble therein, and thus was not characterized electrochemically. The cyclic voltammograms (CV) of compound **2** at multiple scan rates are shown in Fig. 3 ▸. The main feature presented by compound **2** exhibits a redox couple at *ca* −0.5 V *vs F*_c_/*F*_c_^+^ associated with the Cu^2+^/Cu^1+^ couple (Iqbal *et al.*, 2013 ▸; Bonomo *et al.*, 2000 ▸). This redox process was found to be quasi-reversible because as the scan rate increased, the peak-to-peak separation increased, indicating that this process is not reversible. Another indication of the quasi-reversible nature of compound **2** is that the ratio between the cathodic and anodic peak current is less than 1. According to the Randles–Sevcik equation, the observed linear relationship between the square root of the scan rate and the peak current confirms that the quasi-revers­ible process is diffusion-controlled (Fig. 4 ▸) (Elgrishi *et al.*, 2018 ▸). It was observed that on increasing the scan rates to 750 mV s^−1^, two irreversible oxidation processes appeared at 0.05 and 0.50 V *vs F*_c_/*F*_c_^+^. In summary, complex **2** possesses a quasi-reversible diffusion-controlled redox process corres­ponding to the Cu^2+^/Cu^1+^ couple.

## 5. Hirshfeld surface analysis

**Figure 5:**
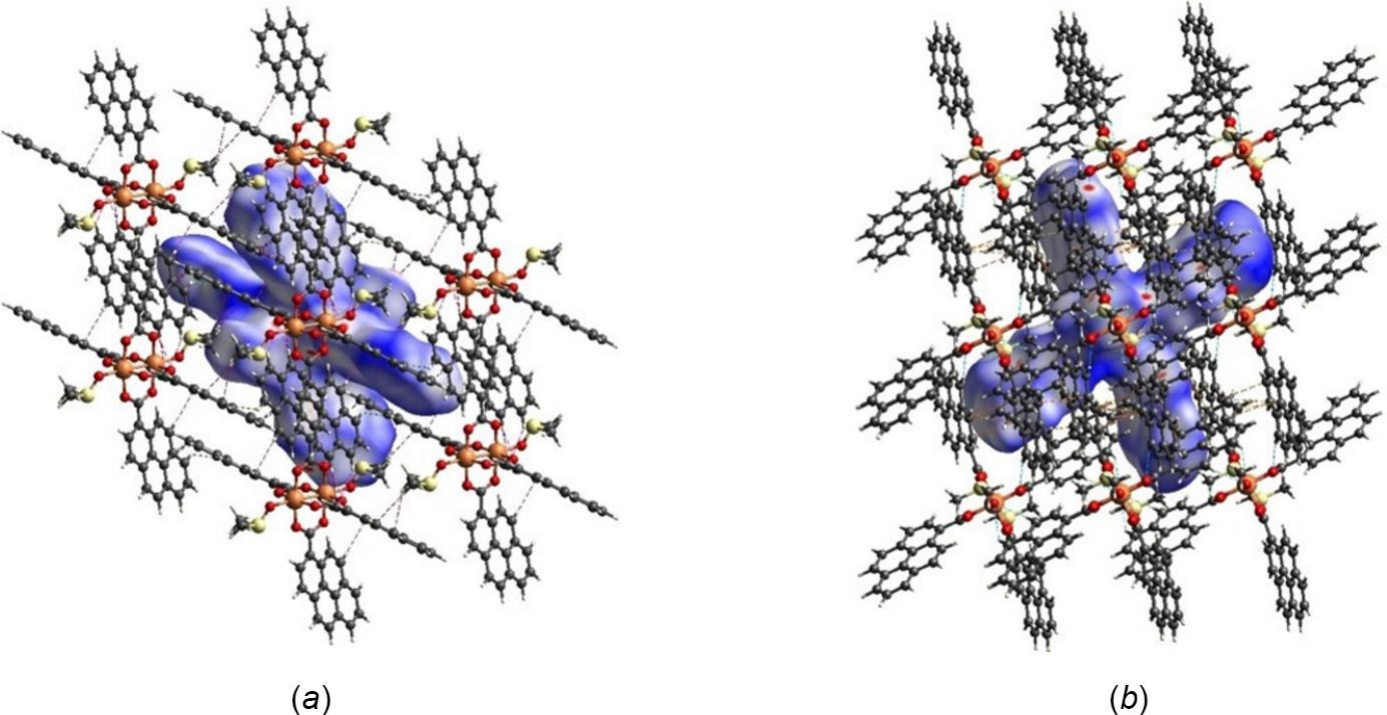
Hirshfeld surface evaluated over *d*_norm_ for title compound **1** with adjacent mol­ecules showing short contacts (*a*) C⋯H/H⋯C (purple) and H⋯H (green), (*b*) H⋯O/O⋯H (blue) and C⋯C (orange).

The Hirshfeld surfaces were generated using *CrystalExplorer17.5* software and evaluated over *d*_norm_, shape-index, and curvedness. Fingerprint plot analysis was also carried out for **1** and **2** (Spackman *et al.*, 2021 ▸; Turner *et al.*, 2017 ▸; Spackman & Jayatilaka, 2009 ▸; Spackman & McKinnon, 2002 ▸). The Hirshfeld surface of **1** evaluated over *d*_norm_ shows multiple bright- and light-red spots (Figs. 5 ▸ and 6 ▸), revealing that many inter­actions take place and that the crystal packing is a compact one (*i.e*., short distances). The red spots on the surface, including the innermost region near the oxygen atoms (*e.g.*, O1⋯H34 at 2.53 Å), equatorial pyrene moieties (core and edges, *e.g.*, C28⋯H15 at 2.87 Å, H32⋯H9 at 2.38 Å), and axial ligand positions (*e.g.*, O5⋯H4 at 2.56 Å, H36*B*⋯C11 at 2.77 Å), are mostly C⋯C, H⋯H, H⋯O/O⋯H, and C⋯H/H⋯C inter­actions (Fig. 5 ▸). The red spots on the surface region of axial ligands indicate that inter­actions with DMSO are a crucial component for the crystal packing.

**Figure 6:**
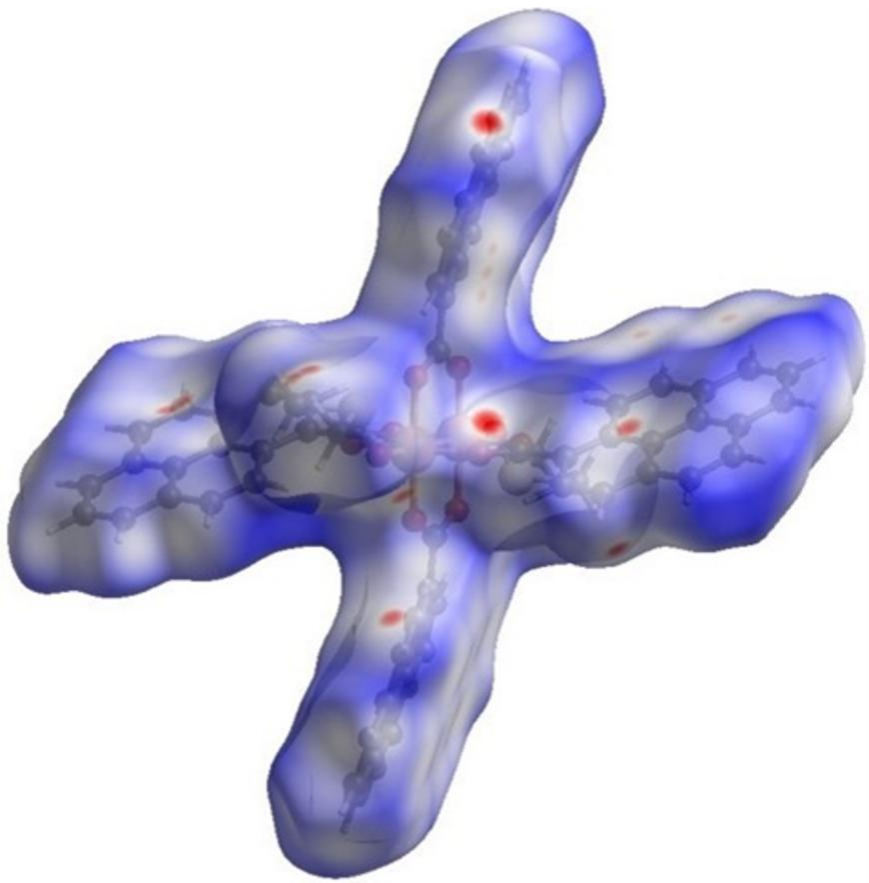
Hirshfeld surface evaluated over *d*_norm_ for title compound **1**.

**Figure 7:**
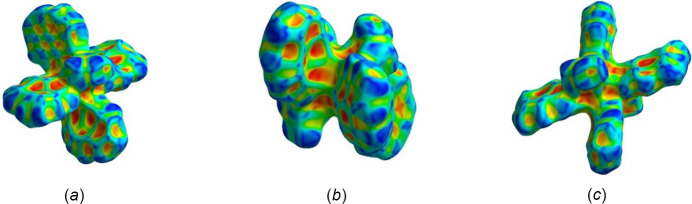
Hirshfeld surface evaluated over shape-index for title compound **1**, viewed from the side (*a*), (*b*) and top (*c*).

**Figure 8:**
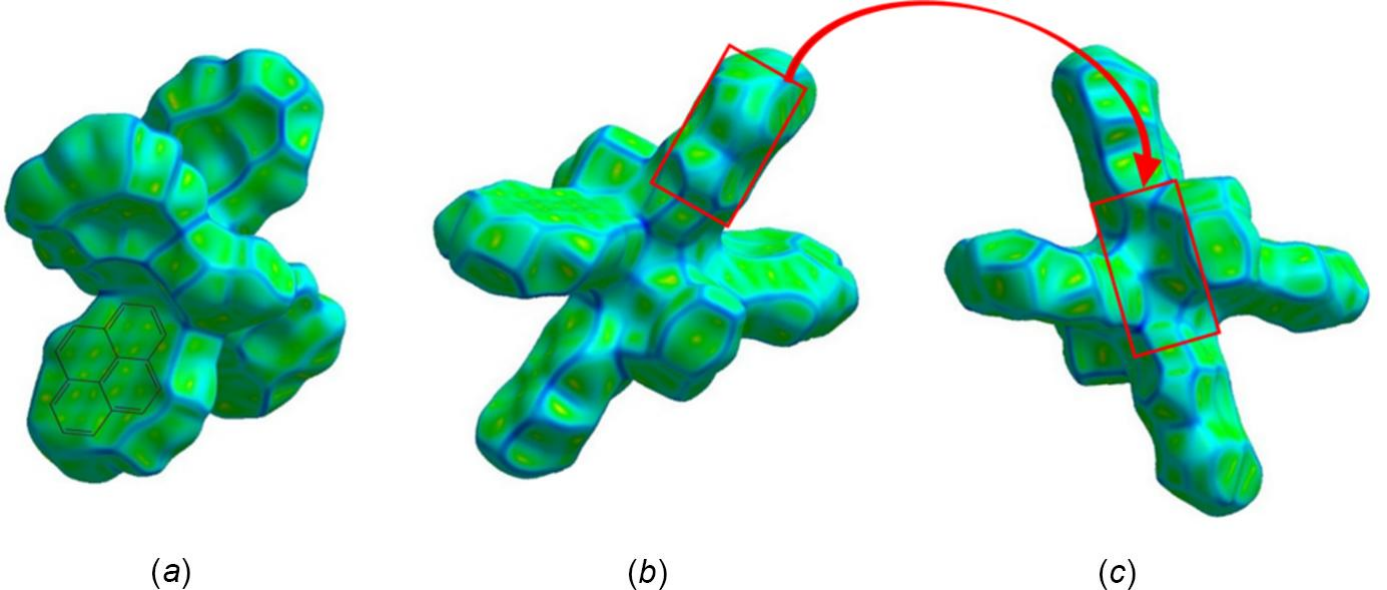
Hirshfeld surface evaluated over curvedness for title compound **1**, viewed from the side (*a*) and top (*b*), (*c*).

Short inter­actions are better perceived in Fig. 6 ▸, both on the pyrene moiety (core and edges) and ligand positions. The role of pyrene rings in the crystal packing is evident, as well as for solvent mol­ecules, even though their contributions are different (Fig. 7 ▸). Important π–π inter­actions are observed in the shape-index surface, represented by characteristic adjacent red–yellow and blue–green triangles (and back-to-back diamonds) on pyrene rings (Fig. 7 ▸ *a*) and in the axial ligand region (Fig. 7 ▸ *c*). Inter­estingly, not all pyrene cores have the same degree of inter­actions within the crystal packing. The pyrene rings are either engaged in strong π–π inter­actions or in other inter­actions, predominantly of C⋯H/H⋯C(core) and H⋯H(edges) type. The inter­centroid distance for rings that exhibit strong π–π inter­actions is 3.75 Å and these rings greatly overlap. Fig. 7 ▸ *b* defines hollows toward the center of the mol­ecule and bumps on the pyrene edges, confirming that inter­molecular inter­actions allow mol­ecules to inter­lock for the crystal packing,

Evaluation of the curvedness reflects the planarity of the pyrene rings, specifically for those exhibiting strong π–π inter­actions (Fig. 8 ▸ *a*), while the other rings and solvent ligands have both flat and positive curvatures. Hence, compound **1** has diverse inter­actions that give way to the resulting array. Fig. 8 ▸ *a* portrays a superposition of where the pyrene ring is located below the generated surface; only a green color (*i.e*., flatness) is observed in this region. Going from Fig. 8 ▸ *b* to Fig. 8 ▸ *c*, the red boxes are localized to fit together inter­locking pyrene moieties towards the innermost region of the mol­ecule, highlighting complementarity in the crystal-packing array.

**Figure 9:**
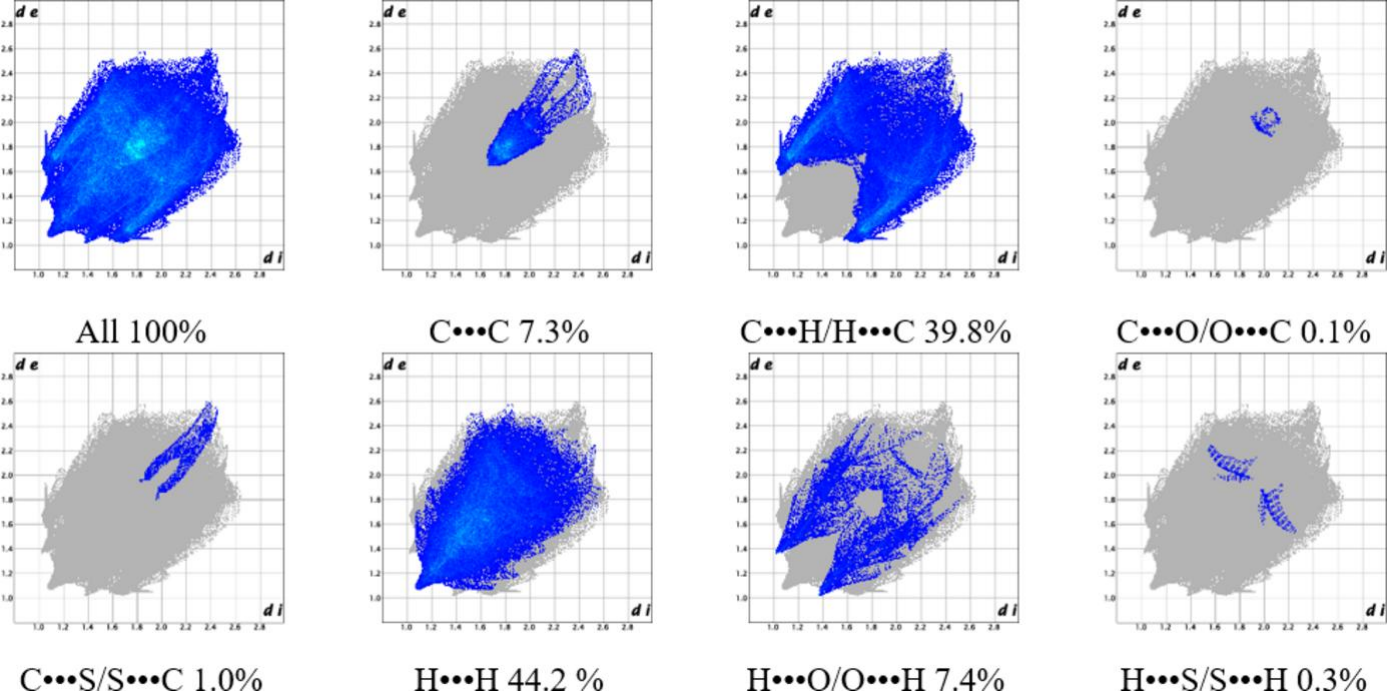
Fingerprint plot analysis for title compound **1**.

The fingerprint plot for compound **1** is symmetric, and contacts occur over a long range of distances (*i.e*., *d*_e_ and *d*_i_ scale) for C⋯H/H⋯C (39.8%), H⋯H (44.2%), and H⋯O/O⋯H (7.4%) type primarily. H⋯H contacts make up almost half the total inter­actions (44.2%). A large concentration of points is centered around 1.6–2.0 Å, linked to π–π stacking; contacts of C⋯C (7.3%) type comprise DMSO–pyrene and pyrene–pyrene. Furthermore, characteristic traits are distinguished: both peaks and wings are demarcated in the C⋯H/H⋯C, H⋯H, and H⋯O/O⋯H plots, so different contacts are present; they all incorporate the pyrene moieties and solvent ligands. Upon further analysis, it was found that DMSO participates in each type of contact in Fig. 9 ▸, either from the sulfur, oxygen, or methyl groups. Contacts of the C⋯O/O⋯C (0.1%), C⋯S/S⋯C (1.0%), and H⋯S/S⋯H (0.3%) types are not made out from short-contact inter­action analysis because their distances are very long. Contacts of the C⋯O/O⋯C type arise from carboxyl­ate and DMSO oxygen atoms to pyrene ring carbons, the C⋯S/S⋯C type go from the DMSO sulfur atom to pyrene ring carbons, and H⋯S/S⋯H from the DMSO sulfur atom to pyrene ring hydrogens.

**Figure 10:**
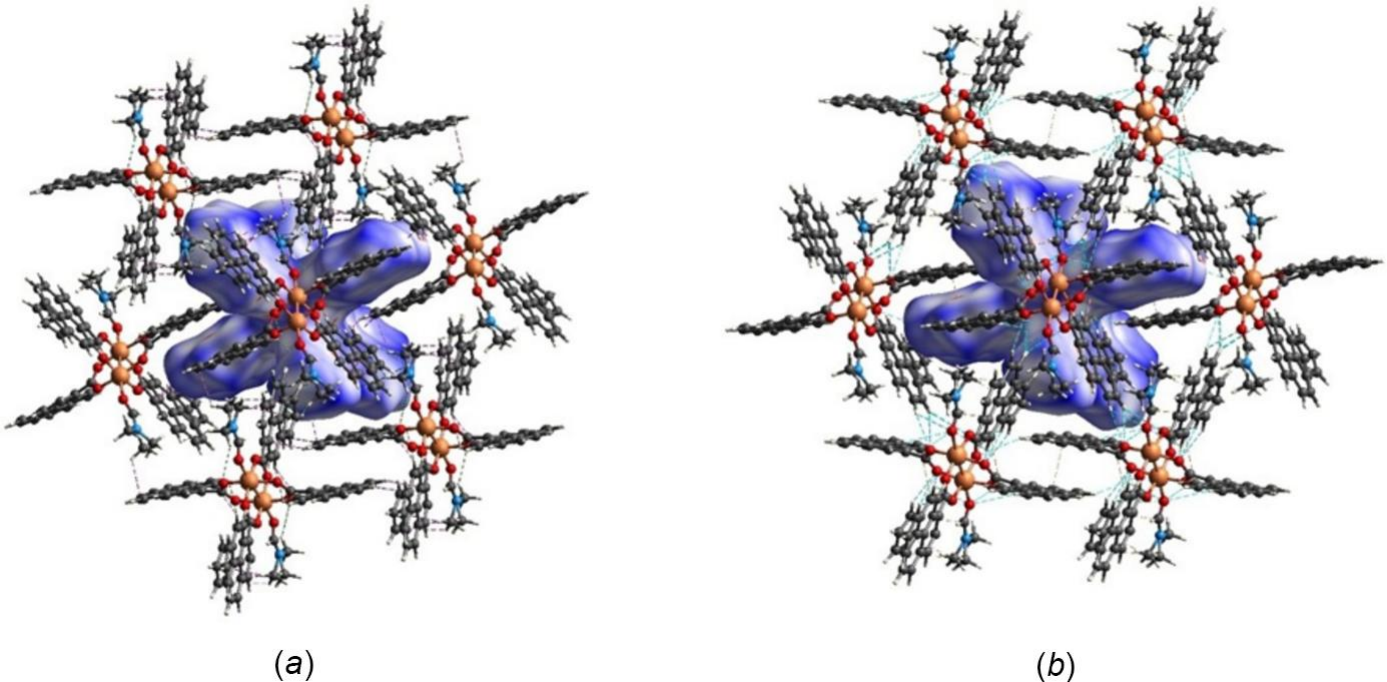
Hirshfeld surface evaluated over *d*_norm_ for title compound **2** with adjacent mol­ecules showing short contacts (*a*) C⋯H/H⋯C (purple) and H⋯H (green), (*b*) H⋯O/O⋯H (blue) and C⋯C (orange).

The Hirshfeld surface generated for title compound **2** evaluated over *d*_norm_ shows the significance of the axial ligands as well as the pyrene moieties, like in compound **1**. As seen in Fig. 10 ▸, adjacent mol­ecules surrounding the generated surface deliver multiple inter­actions, which are distributed from the innermost region near the oxygen atoms (*e.g.*, O2⋯H34*A* at 2.79 Å, O2⋯H34*B* at 2.61 Å), equatorial pyrene moieties (core and edges) (*e.g.*, C4⋯C4 at 3.18 Å, C24⋯H16 at 2.70 Å), to the axial ligands (*e.g*., H37*B*⋯C16 at 2.87 Å).

**Figure 11:**
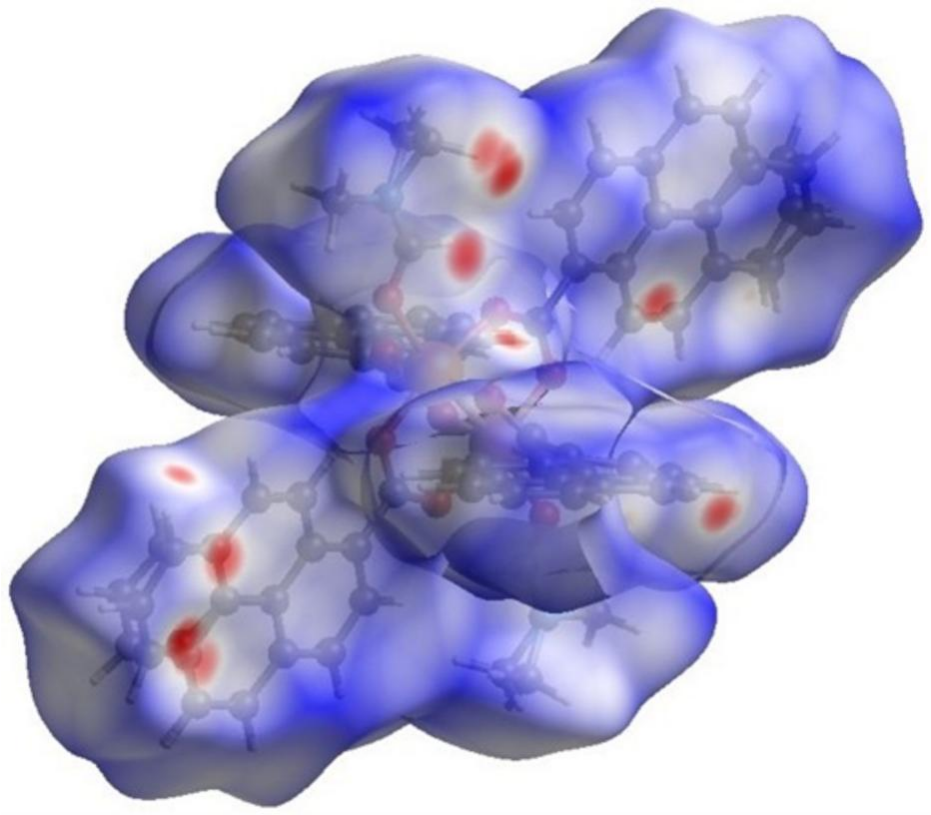
Hirshfeld surface evaluated over *d*_norm_ for title compound **2**.

Most of the red spots are intense (*i.e*., short distance), mainly C⋯C, H⋯H, H⋯O/O⋯H, and C⋯H/H⋯C type inter­actions. However, just a few light-red spots (*i.e*., longer distance) color are recognized as additional contacts, primarily C⋯H/H⋯C type. In Fig. 11 ▸, a few red spots are present on the pyrene aromatic core and most are located near the edges. In contrast, the DMF region has strong red spots. When comparing Fig. 6 ▸ and Fig. 11 ▸, the latter surface contains a qualitatively greater amount of blue regions; however, the red spots are more intense, implying compound **1** has strong inter­actions distributed over more parts of the surface but compound **2** has shorter distances in most of its inter­actions.

**Figure 12:**
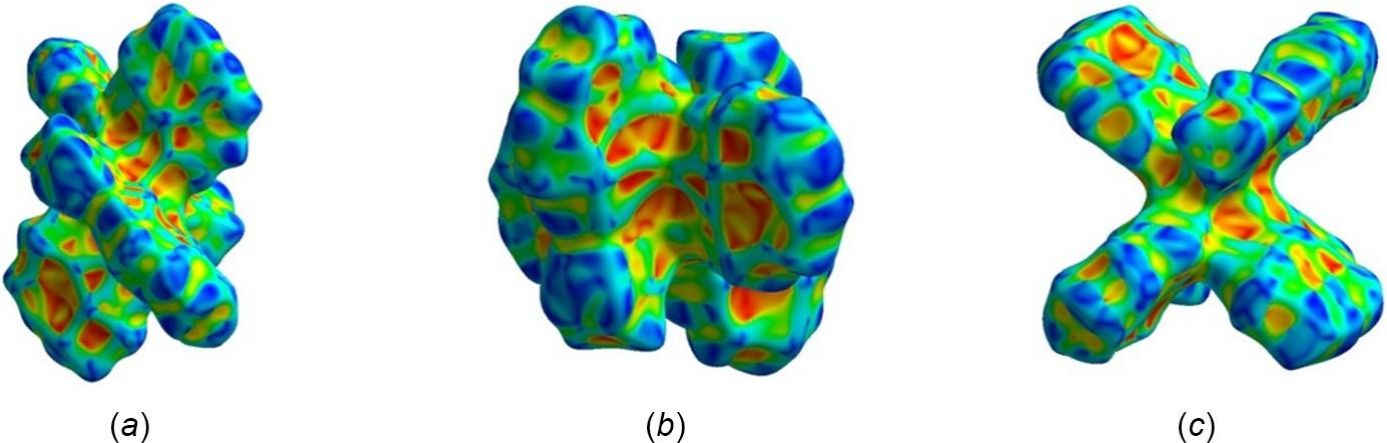
Hirshfeld surface evaluated over shape-index for title compound **2**, viewed from the side (*a*), (*b*) and top (*c*).

Pyrene ring surfaces with red–yellow and blue–green adjacent triangles, as displayed in Fig. 12 ▸ *a*, are characteristic of π–π inter­actions, which are expected due to the nature of the PAHs. Similar to compound **1**, not all rings show this degree of inter­action because of the position of each pyrene ring with respect to other moieties in the crystal-packing array and corresponding inter­actions. Different from Fig. 7 ▸ *a*, Fig. 12 ▸ *a* has a less uniform pattern of π–π inter­actions than for title compound **1**, as a result of the less overlapping pyrene rings. Rings with weak π–π inter­actions have more C⋯H/H⋯C(core) and H⋯H(edges) contacts, analogous to compound **1**.

**Figure 13:**
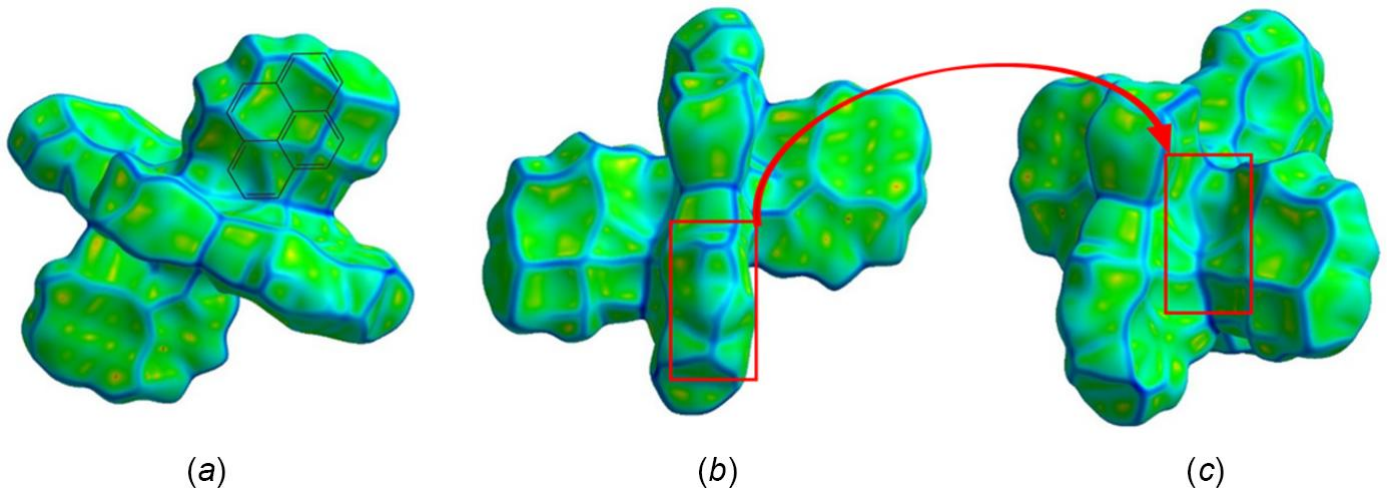
Hirshfeld surface evaluated over curvedness for title compound **2**, viewed from the side (*a*) and top (*b*), (*c*).

The planarity of the pyrene moieties is depicted by the curvedness (Fig. 13 ▸ *a*) where most of the surface is flat. However, even pyrene rings that exhibit strong π–π inter­actions do not possess a completely flat surface region (unlike in compound **1**), and the other rings have alternating regions of flatness. The inter­centroid distance for rings that exhibit strong π–π inter­actions is 5.83 Å, farther apart than for compound **1**. In addition, the red box in Fig. 13 ▸ *b* can be translated into the one in Fig. 13 ▸ *c*; thus, complementarity is observed within the generated surface, coming from inter­molecular inter­actions that follow the screw axes and glide planes present in title compound **2** (*P*2_1_/*n*). Both compounds achieve complementarity in their crystal packing, but each arises from different inter­molecular inter­actions.

**Figure 14:**
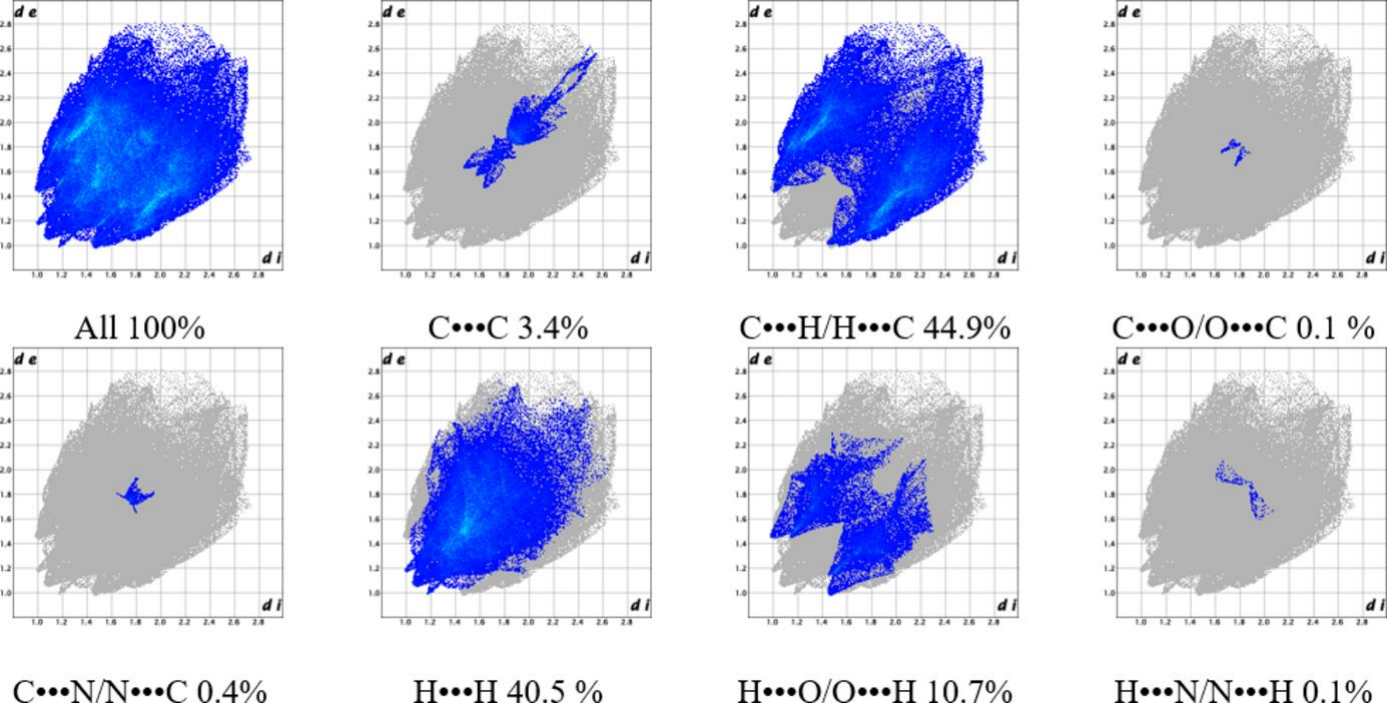
Fingerprint plot analysis for title compound **2**.

The 2D fingerprint plot for compound **2** (Fig. 14 ▸) has the following features: it is quasi-symmetric, C⋯H/H⋯C inter­actions account for almost half of the contacts (44.9%) followed by H⋯H (40.5%), with fewer contributions from H⋯O/O⋯H (10.7%) and C⋯C (3.4%) inter­actions. C⋯H/H⋯C contacts have broad peaks spread out over most of the plot, H⋯H contacts also cover a broad range of distances and several types of inter­actions, and H⋯O/O⋯H contacts have wide peaks and fewer weak contacts. In the same way as for compound **1**, all contacts in Fig. 14 ▸ include atoms from the solvent, DMF in the case of compound **2**. Likewise, contacts of the C⋯O/O⋯C (0.1%), C⋯N/N⋯C (0.4%), and H⋯N/N⋯H (0.1%) types are not identified from short-contact inter­action analysis because the distances are long. Contacts of the C⋯O/O⋯C type arise from carboxyl­ate and DMF oxygen atoms to pyrene ring carbons (as in compound **1**), the C⋯N/N⋯C type go from the DMF nitro­gen atom to pyrene ring carbons, and H⋯N/N⋯H from the DMF nitro­gen atom to pyrene ring hydrogens.

## 6. Database survey

A search of the Cambridge Structural Database (CSD Version 5.44, June 2023 update; Groom *et al.*, 2016 ▸) for the two reported compounds revealed a total of five hits containing polycyclic aromatic copper dimers. None of the these was an exact match to the pyrene moieties of the title compounds. Four of them included naphthalene moieties and the remaining structure contained phenanthrene. The four structures that contained naphthalene groups are *catena*-[tetra­kis­(μ_4_-naphthalene-2,6-di­carboxyl­ato)bis­(μ_2_-4,4′-bi­pyridine)­tet­ra­copper(II) bis­(μ_4_-naphthalene-2,6-di­carboxyl­ato)(μ_2_-4,4′-bi­pyridine)­dicopper(II)] (BUSQOW; Kanoo *et al.*, 2009 ▸), tetra­kis­(μ-naphthalene-2-carboxyl­ato)bis­(aceto­nitrile)­dicop­per aceto­nitrile solvate (CUJFAR; Liu *et al.*, 2020 ▸), tetra­kis­(μ-2-naphtho­ato)bis­(aceto­nitrile)­dicopper aceto­nitrile solvate (WUNRII; Goldberg *et al.*, 2015 ▸), and tetra­kis­(μ-2-naphtho­ato)bis­(2,3-di­methyl­pyridine)­dicopper (WUNROO; Goldberg *et al.*, 2015 ▸). The axial ligands present in CUJFAR, WUNRII, and WUNROO are involved in inter­molecular inter­actions with adjacent mol­ecules that contribute to the crystal-packing array. The naphthalene ligands in the above-mentioned structures participate in π–π inter­actions. Moreover, the nature of the axial ligands determines the contribution of π inter­actions to the crystal packing. For example, in WUNROO, the properties and position of the 2,3-lutidine (aromatic heterocycle mol­ecule) allowed for enhanced π inter­actions. In contrast, CUJFAR and WUNRII contain aceto­nitrile (non-aromatic linear mol­ecule) as their axial ligand and present mainly C⋯C and C⋯H inter­actions. Finally, the last hit, corresponding to tetra­kis­(μ_2_-phenanthrene-9-carboxyl­ato)bis­(*N*,*N*-di­methyl­formamide)­dicopper(II) (WUZCEA; Wang *et al.*, 2010 ▸) resembles title compound **2** in having a phenanthrene instead of a pyrene equatorial ligand, resulting in a change of the space-group setting from *P*2_1_/*c* (WUZCEA) to *P*2_1_/*n* (**2**). In terms of the packing structure, WUZCEA exhibits fewer short contacts, C⋯C and O⋯H type inter­actions than compound **2**, which presents C⋯C, H⋯H, C⋯H, and O⋯H type inter­actions. Nonetheless, WUZCEA exhibits more π–π inter­actions than compound **2**. Similarly to the other compounds reported in this survey, in WUZCEA the axial ligands play an important role in the crystal packing of the mol­ecules.

## 7. Synthesis and crystallization

All the chemicals were purchased from Sigma-Aldrich. The chemicals and solvents were used as supplied without further purification. IR spectra were recorded on a FT–IR Frontier Perkin Elmer spectrophotometer with ATR modality in the region 4000–600 cm^−1^. UV–vis spectra were recorded on a UV-1900 spectrophotometer in the range 200–1000 nm using a 1 cm path-length cell for solution in DMSO or DMF. The CVs were recorded in a BioLogic potentiostat using a solution of 0.1 *M* TBAPF_6_ with a glassy carbon working electrode, a graphite rod counter-electrode, and a 0.01 *M* AgNO_3_ silver wire pseudo-reference electrode corrected with ferrocene.

Synthesis of [Cu_2_(pyr-COO^−^)_4_(DMSO)_2_] (**1**):

1-Pyrene carb­oxy­lic acid (0.084 g, 0.3 mmol) was dissolved in 18 mL of methanol and deprotonated with tri­ethyl­amine (0.034 g, 0.23 mmol). The pyrene-1-carboxyl­ate solution was added slowly to a methano­lic solution of Cu(NO_3_)_2_·H_2_O (0.0819 g, 0.033 mmol) at room temperature, which afforded a green solid. The mixture was stirred for 24 h and was then filtered out. The solid was dissolved in DMSO for crystallization. Single crystals were obtained by vapor diffusion of methanol into dimethyl sulfoxide after one week. Yield: (85.6 mg, 68%). IR (ATR); *ν* (cm^−1^) : 3041 (*w*), 1920 (*w*), 1672 (*w*), 1589 (*s*), 1506 (*m*), 1392 (*s*), 1359 (*s*), 1312 (*m*), 1165 (*m*), 838 (*s*), 710 (*s*), 619 (*m*). UV–vis; *λ*_max_ (DMSO, nm) 280 (pyr–COO) (Niko *et al.*, 2012 ▸; Johnpeter & Therrien, 2013 ▸), 335 (pyr–COO, π–π* transition) (Haldar *et al.*, 2016 ▸), 352 (pyr–COO) (Niko *et al.*, 2012 ▸; Johnpeter & Therrien, 2013 ▸), 379 (pyr–COO) (Niko *et al.*, 2012 ▸; Johnpeter & Therrien, 2013 ▸), and 739 (Cu, *d*–*d* and MLCT transitions) (Wang *et al.*, 2021 ▸).

Synthesis of [Cu_2_(pyr-COO^−^)_4_(DMF)_2_] (**2**):

A similar synthetic procedure as for **1** was used. However, the crystallization process was different. The resulting solid was dissolved in DMF for crystallization. Single crystals were obtained by vapor diffusion of methanol into dimethyl formamide after one week. Yield: (87.1 mg, 69%). IR (ATR); *ν* (cm^−1^): 1655 (*m*), 1607(*s*), 1591 (*s*), 1385 (*s*), 1359 (*s*), 1314 (*m*), 1165 (*m*), 853 (*s*), 820 (*s*), 760 (*s*), 671 (*m*). UV–vis; *λ*_max_ (DMF, nm) 283 (pyr–COO) (Niko *et al.*, 2012 ▸; Johnpeter & Therrien, 2013 ▸), 337 (pyr–COO, π-π-* transitions) (Haldar *et al.*, 2016 ▸), 352 (pyr–COO) (Niko *et al.*, 2012 ▸; Johnpeter & Therrien, 2013 ▸), 382 (pyr–COO) (Niko *et al.*, 2012 ▸; Johnpeter & Therrien, 2013 ▸), and 701 (Cu, *d*–*d* and MLCT transitions) (Wang *et al.*, 2021 ▸).

## 8. Refinement

Crystal data, data collection and structure refinement details are summarized in Table 1 ▸. H atoms were included in geometrically calculated positions and refined as riding atoms with C—H = 0.93 Å and *U*_iso_(H) = *1.2U*_eq_(C).

## Supplementary Material

Crystal structure: contains datablock(s) 1, 2. DOI: 10.1107/S2056989023010277/ny2001sup1.cif

Structure factors: contains datablock(s) 1. DOI: 10.1107/S2056989023010277/ny20011sup2.hkl

Structure factors: contains datablock(s) 2. DOI: 10.1107/S2056989023010277/ny20012sup3.hkl

CCDC references: 2281680, 2281679

Additional supporting information:  crystallographic information; 3D view; checkCIF report

## Figures and Tables

**Table 1:** Experimental details

	**1**	**2**
Crystal data		
Chemical formula	[Cu_2_(C_17_H_9_O_2_)_4_(C_2_H_6_OS)_2_]	[Cu_2_(C_17_H_9_O_2_)_4_(C_3_H_7_NO)_2_]
*M*_r_	1264.30	1254.24
Crystal system, space group	Triclinic, *P* 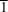	Monoclinic, *P*2_1_/*n*
Temperature (K)	100	300
*a*, *b*, *c* (Å)	10.5283 (1), 11.8583 (2), 11.9401 (1)	10.54266 (13), 21.8888 (2), 12.66517 (15)
α, β, γ (°)	101.044 (1), 98.136 (1), 104.142 (1)	90, 100.1160 (11), 90
*V* (Å^3^)	1390.69 (3)	2877.25 (6)
*Z*	1	2
Radiation type	Cu *K*α	Cu *K*α
μ (mm^−1^)	2.19	1.46
Crystal size (mm)	0.12 × 0.11 × 0.09	0.12 × 0.08 × 0.04

Data collection		
Diffractometer	Rigaku SuperNova Hypix6000	Rigaku SuperNova Hypix6000
Absorption correction	Multi-scan (Blessing, 1995 ▸)	Multi-scan (Blessing, 1995 ▸)
*T*_min_, *T*_max_	0.778, 0.818	0.844, 0.944
No. of measured, independent and observed [*I* > 2σ(*I*)] reflections	78157, 5097, 4827	15433, 5246, 4485
*R*_int_	0.048	0.023
(sin θ/λ)_max_ (Å^−1^)	0.604	0.603

Refinement		
*R*[*F* ^2^ > 2σ(*F* ^2^)], *wR*(*F* ^2^), *S*	0.032, 0.082, 1.05	0.031, 0.091, 1.07
No. of reflections	5142	5246
No. of parameters	391	447
No. of restraints	0	1
H-atom treatment	H-atom parameters constrained	H-atom parameters constrained
Δρ_max_, Δρ_min_ (e Å^−3^)	0.45, −0.47	0.20, −0.28

Computer programs: *CrysAlis PRO* (Rigaku OD, 2020 ▸), *SHELXT* (Sheldrick, 2015*a* ▸), *SHELXL2018/3* (Sheldrick, 2015*b* ▸), and *OLEX2* (Dolomanov *et al.*, 2009 ▸).

## supplementary crystallographic information

### Bis(dimethyl sulfoxide)tetrakis(µ-pyrene-1-carboxylato)dicopper(Cu—Cu) (1) Crystal data

**Table:**

[Cu_2_(C_17_H_9_O_2_)_4_(C_2_H_6_OS)_2_]	*Z* = 1
*M**_r_* = 1264.30	*F*(000) = 650
Triclinic, *P*1	*D*_x_ = 1.510 Mg m^−^^3^
*a* = 10.5283 (1) Å	Cu *K*α radiation, λ = 1.54184 Å
*b* = 11.8583 (2) Å	Cell parameters from 5097 reflections
*c* = 11.9401 (1) Å	θ = 0.8–0.8°
α = 101.044 (1)°	µ = 2.19 mm^−^^1^
β = 98.136 (1)°	*T* = 100 K
γ = 104.142 (1)°	Block, green
*V* = 1390.69 (3) Å^3^	0.12 × 0.11 × 0.09 mm

### Bis(dimethyl sulfoxide)tetrakis(µ-pyrene-1-carboxylato)dicopper(Cu—Cu) (1) Data collection

**Table:**

Rigaku SuperNova Hypix6000 diffractometer	5097 independent reflections
Radiation source: microsource	4827 reflections with *I* > 2σ(*I*)
Detector resolution: 10.0000 pixels mm^-1^	*R*_int_ = 0.048
ω scans	θ_max_ = 68.6°, θ_min_ = 3.9°
Absorption correction: multi-scan (Blessing, 1995)	*h* = −12→12
*T*_min_ = 0.778, *T*_max_ = 0.818	*k* = −11→14
78157 measured reflections	*l* = −14→14

### Bis(dimethyl sulfoxide)tetrakis(µ-pyrene-1-carboxylato)dicopper(Cu—Cu) (1) Refinement

**Table:**

Refinement on *F*^2^	Hydrogen site location: inferred from neighbouring sites
Least-squares matrix: full	H-atom parameters constrained
*R*[*F*^2^ > 2σ(*F*^2^)] = 0.032	*w* = 1/[σ^2^(*F*_o_^2^) + (0.0369*P*)^2^ + 1.3914*P*] where *P* = (*F*_o_^2^ + 2*F*_c_^2^)/3
*wR*(*F*^2^) = 0.082	(Δ/σ)_max_ = 0.001
*S* = 1.05	Δρ_max_ = 0.45 e Å^−^^3^
5142 reflections	Δρ_min_ = −0.46 e Å^−^^3^
391 parameters	Extinction correction: *SHELXL2018/3* (Sheldrick, 2015b), Fc^*^=kFc[1+0.001xFc^2^λ^3^/sin(2θ)]^-1/4^
0 restraints	Extinction coefficient: 0.00045 (11)

### Bis(dimethyl sulfoxide)tetrakis(µ-pyrene-1-carboxylato)dicopper(Cu—Cu) (1) Special details

**Table:**

Geometry. All esds (except the esd in the dihedral angle between two l.s. planes) are estimated using the full covariance matrix. The cell esds are taken into account individually in the estimation of esds in distances, angles and torsion angles; correlations between esds in cell parameters are only used when they are defined by crystal symmetry. An approximate (isotropic) treatment of cell esds is used for estimating esds involving l.s. planes.

### Bis(dimethyl sulfoxide)tetrakis(µ-pyrene-1-carboxylato)dicopper(Cu—Cu) (1) Fractional atomic coordinates and isotropic or equivalent isotropic displacement parameters (Å^2^)

**Table:**

	*x*	*y*	*z*	*U*_iso_*/*U*_eq_	
Cu1	0.91294 (2)	0.49438 (2)	0.40919 (2)	0.01418 (9)	
S3	0.79851 (5)	0.59369 (4)	0.19711 (4)	0.02605 (13)	
O1	0.82175 (13)	0.56000 (12)	0.52641 (11)	0.0228 (3)	
O2	0.96841 (13)	0.56908 (13)	0.68455 (11)	0.0257 (3)	
O3	0.82877 (13)	0.33234 (11)	0.42304 (11)	0.0229 (3)	
O4	0.97457 (13)	0.34410 (12)	0.58282 (12)	0.0236 (3)	
O5	0.76843 (12)	0.49904 (12)	0.26558 (11)	0.0223 (3)	
C1	0.86206 (17)	0.58347 (16)	0.63416 (16)	0.0172 (4)	
C2	0.77238 (18)	0.63064 (16)	0.70712 (16)	0.0179 (4)	
C3	0.63637 (18)	0.59548 (17)	0.65730 (16)	0.0199 (4)	
H3	0.606849	0.545289	0.580998	0.024*	
C4	0.54310 (18)	0.63170 (16)	0.71598 (17)	0.0199 (4)	
H4	0.450794	0.603095	0.681218	0.024*	
C5	0.58426 (19)	0.70972 (17)	0.82549 (16)	0.0207 (4)	
C6	0.72345 (19)	0.74971 (17)	0.87799 (16)	0.0193 (4)	
C7	0.81763 (18)	0.70674 (17)	0.81909 (15)	0.0186 (4)	
C8	0.95692 (19)	0.74954 (19)	0.87781 (17)	0.0256 (4)	
H8	1.022196	0.723354	0.840727	0.031*	
C9	0.9957 (2)	0.8256 (2)	0.98378 (17)	0.0294 (5)	
H9	1.087549	0.850467	1.019974	0.035*	
C10	0.9021 (2)	0.8703 (2)	1.04378 (17)	0.0281 (4)	
C11	0.76560 (19)	0.83241 (18)	0.98852 (16)	0.0222 (4)	
C12	0.6704 (2)	0.87714 (18)	1.04386 (17)	0.0237 (4)	
C13	0.5324 (2)	0.83475 (19)	0.98846 (18)	0.0276 (4)	
H13	0.468633	0.863931	1.025241	0.033*	
C14	0.4911 (2)	0.75446 (18)	0.88537 (18)	0.0260 (4)	
H14	0.398478	0.726802	0.851516	0.031*	
C15	0.7158 (2)	0.9604 (2)	1.15152 (18)	0.0302 (5)	
H15	0.653921	0.993068	1.187969	0.036*	
C16	0.8488 (2)	0.9958 (2)	1.20560 (18)	0.0360 (5)	
H16	0.877114	1.051076	1.279330	0.043*	
C17	0.9414 (2)	0.9512 (2)	1.15288 (18)	0.0357 (5)	
H17	1.032500	0.975761	1.191211	0.043*	
C18	0.87448 (16)	0.28873 (16)	0.50314 (15)	0.0154 (4)	
C19	0.81254 (17)	0.15973 (16)	0.50049 (15)	0.0153 (3)	
C20	0.89333 (18)	0.10243 (17)	0.55814 (15)	0.0186 (4)	
H20	0.976743	0.148747	0.606131	0.022*	
C21	0.85498 (18)	−0.01959 (17)	0.54708 (16)	0.0194 (4)	
H21	0.911828	−0.055685	0.587996	0.023*	
C22	0.73365 (18)	−0.09086 (16)	0.47652 (15)	0.0180 (4)	
C23	0.64539 (17)	−0.03391 (16)	0.42348 (15)	0.0154 (4)	
C24	0.68319 (17)	0.09253 (16)	0.43646 (14)	0.0147 (3)	
C25	0.58634 (17)	0.14480 (16)	0.38548 (15)	0.0169 (4)	
H25	0.607986	0.229155	0.395251	0.020*	
C26	0.46483 (17)	0.07596 (17)	0.32380 (15)	0.0180 (4)	
H26	0.402257	0.113635	0.293537	0.022*	
C27	0.42811 (17)	−0.05156 (17)	0.30298 (15)	0.0183 (4)	
C28	0.51893 (17)	−0.10641 (16)	0.35411 (15)	0.0170 (4)	
C29	0.48514 (19)	−0.23307 (17)	0.33528 (16)	0.0215 (4)	
C30	0.3614 (2)	−0.30173 (18)	0.26544 (18)	0.0278 (4)	
H30	0.337925	−0.386475	0.252135	0.033*	
C31	0.2734 (2)	−0.24735 (19)	0.21585 (18)	0.0289 (5)	
H31	0.190048	−0.295055	0.168667	0.035*	
C32	0.30546 (19)	−0.12393 (18)	0.23425 (17)	0.0239 (4)	
H32	0.243618	−0.087873	0.199899	0.029*	
C33	0.5800 (2)	−0.28605 (17)	0.38834 (18)	0.0258 (4)	
H33	0.559200	−0.370679	0.375341	0.031*	
C34	0.6964 (2)	−0.21894 (17)	0.45530 (17)	0.0236 (4)	
H34	0.756188	−0.256821	0.489781	0.028*	
C35	0.8858 (3)	0.5371 (3)	0.0929 (2)	0.0514 (7)	
H35A	0.970862	0.530951	0.132917	0.077*	
H35B	0.902953	0.591272	0.040810	0.077*	
H35C	0.831661	0.457693	0.047304	0.077*	
C36	0.6437 (2)	0.5714 (2)	0.10162 (19)	0.0337 (5)	
H36A	0.605648	0.486001	0.064883	0.051*	
H36B	0.658719	0.616149	0.041424	0.051*	
H36C	0.581536	0.599821	0.146035	0.051*	

### Bis(dimethyl sulfoxide)tetrakis(µ-pyrene-1-carboxylato)dicopper(Cu—Cu) (1) Atomic displacement parameters (Å^2^)

**Table:**

	*U*^11^	*U*^22^	*U*^33^	*U*^12^	*U*^13^	*U*^23^
Cu1	0.01343 (14)	0.01139 (15)	0.01692 (14)	0.00270 (10)	0.00178 (10)	0.00332 (10)
S3	0.0222 (2)	0.0246 (3)	0.0284 (3)	0.00069 (19)	−0.00273 (18)	0.0127 (2)
O1	0.0253 (7)	0.0281 (8)	0.0213 (7)	0.0164 (6)	0.0077 (5)	0.0066 (6)
O2	0.0232 (7)	0.0341 (8)	0.0196 (6)	0.0117 (6)	0.0046 (5)	0.0008 (6)
O3	0.0241 (7)	0.0138 (7)	0.0260 (7)	−0.0003 (5)	−0.0032 (5)	0.0067 (5)
O4	0.0217 (6)	0.0179 (7)	0.0260 (7)	−0.0020 (5)	−0.0037 (5)	0.0087 (5)
O5	0.0172 (6)	0.0246 (7)	0.0240 (7)	0.0019 (5)	−0.0011 (5)	0.0117 (6)
C1	0.0183 (9)	0.0105 (8)	0.0233 (9)	0.0034 (7)	0.0065 (7)	0.0043 (7)
C2	0.0205 (9)	0.0149 (9)	0.0216 (9)	0.0071 (7)	0.0065 (7)	0.0077 (7)
C3	0.0200 (9)	0.0160 (9)	0.0247 (9)	0.0043 (7)	0.0050 (7)	0.0076 (8)
C4	0.0161 (8)	0.0166 (9)	0.0286 (10)	0.0045 (7)	0.0046 (7)	0.0093 (8)
C5	0.0230 (9)	0.0170 (9)	0.0261 (9)	0.0065 (8)	0.0085 (7)	0.0105 (8)
C6	0.0246 (9)	0.0177 (9)	0.0207 (9)	0.0088 (8)	0.0093 (7)	0.0095 (7)
C7	0.0230 (9)	0.0174 (9)	0.0194 (9)	0.0085 (7)	0.0075 (7)	0.0079 (7)
C8	0.0227 (10)	0.0317 (11)	0.0230 (9)	0.0110 (8)	0.0051 (8)	0.0031 (8)
C9	0.0214 (9)	0.0411 (13)	0.0223 (10)	0.0119 (9)	−0.0006 (8)	−0.0010 (9)
C10	0.0281 (10)	0.0356 (12)	0.0237 (10)	0.0144 (9)	0.0051 (8)	0.0070 (9)
C11	0.0269 (10)	0.0230 (10)	0.0212 (9)	0.0104 (8)	0.0089 (8)	0.0082 (8)
C12	0.0307 (10)	0.0239 (10)	0.0226 (9)	0.0119 (8)	0.0120 (8)	0.0096 (8)
C13	0.0272 (10)	0.0292 (11)	0.0344 (11)	0.0127 (9)	0.0158 (9)	0.0130 (9)
C14	0.0228 (9)	0.0246 (11)	0.0347 (11)	0.0073 (8)	0.0104 (8)	0.0126 (9)
C15	0.0354 (11)	0.0349 (12)	0.0251 (10)	0.0148 (10)	0.0135 (9)	0.0065 (9)
C16	0.0412 (12)	0.0446 (14)	0.0207 (10)	0.0166 (11)	0.0057 (9)	−0.0016 (9)
C17	0.0319 (11)	0.0498 (15)	0.0235 (10)	0.0167 (10)	0.0020 (9)	−0.0007 (10)
C18	0.0149 (8)	0.0150 (9)	0.0174 (8)	0.0058 (7)	0.0062 (7)	0.0022 (7)
C19	0.0171 (8)	0.0128 (9)	0.0160 (8)	0.0044 (7)	0.0052 (7)	0.0019 (7)
C20	0.0168 (8)	0.0188 (9)	0.0199 (9)	0.0053 (7)	0.0027 (7)	0.0035 (7)
C21	0.0199 (9)	0.0199 (10)	0.0225 (9)	0.0099 (7)	0.0051 (7)	0.0080 (8)
C22	0.0219 (9)	0.0158 (9)	0.0199 (9)	0.0079 (7)	0.0086 (7)	0.0061 (7)
C23	0.0173 (8)	0.0143 (9)	0.0157 (8)	0.0049 (7)	0.0066 (7)	0.0033 (7)
C24	0.0167 (8)	0.0136 (9)	0.0150 (8)	0.0050 (7)	0.0065 (6)	0.0028 (7)
C25	0.0192 (8)	0.0133 (9)	0.0201 (9)	0.0058 (7)	0.0063 (7)	0.0052 (7)
C26	0.0175 (8)	0.0201 (10)	0.0206 (9)	0.0087 (7)	0.0061 (7)	0.0082 (7)
C27	0.0172 (8)	0.0198 (10)	0.0176 (8)	0.0036 (7)	0.0055 (7)	0.0041 (7)
C28	0.0184 (8)	0.0151 (9)	0.0179 (8)	0.0039 (7)	0.0071 (7)	0.0036 (7)
C29	0.0239 (9)	0.0166 (10)	0.0232 (9)	0.0032 (8)	0.0071 (7)	0.0038 (8)
C30	0.0294 (10)	0.0152 (10)	0.0331 (11)	−0.0008 (8)	0.0053 (8)	0.0016 (8)
C31	0.0210 (9)	0.0255 (11)	0.0314 (11)	−0.0020 (8)	0.0000 (8)	0.0005 (9)
C32	0.0202 (9)	0.0255 (11)	0.0239 (9)	0.0048 (8)	0.0021 (7)	0.0045 (8)
C33	0.0350 (11)	0.0126 (9)	0.0333 (11)	0.0083 (8)	0.0128 (9)	0.0074 (8)
C34	0.0310 (10)	0.0167 (10)	0.0295 (10)	0.0126 (8)	0.0105 (8)	0.0098 (8)
C35	0.0459 (14)	0.077 (2)	0.0506 (15)	0.0258 (14)	0.0248 (12)	0.0384 (15)
C36	0.0270 (11)	0.0388 (13)	0.0342 (11)	0.0053 (9)	−0.0054 (9)	0.0195 (10)

### Bis(dimethyl sulfoxide)tetrakis(µ-pyrene-1-carboxylato)dicopper(Cu—Cu) (1) Geometric parameters (Å, º)

**Table:**

Cu1—Cu1^i^	2.5934 (5)	C16—H16	0.9500
Cu1—O1	1.9530 (13)	C16—C17	1.387 (3)
Cu1—O2^i^	1.9779 (13)	C17—H17	0.9500
Cu1—O3	1.9586 (13)	C18—C19	1.502 (2)
Cu1—O4^i^	1.9672 (13)	C19—C20	1.398 (3)
Cu1—O5	2.1441 (12)	C19—C24	1.420 (2)
S3—O5	1.5098 (13)	C20—H20	0.9500
S3—C35	1.774 (3)	C20—C21	1.379 (3)
S3—C36	1.782 (2)	C21—H21	0.9500
O1—C1	1.250 (2)	C21—C22	1.397 (3)
O2—C1	1.260 (2)	C22—C23	1.423 (2)
O3—C18	1.263 (2)	C22—C34	1.435 (3)
O4—C18	1.259 (2)	C23—C24	1.426 (3)
C1—C2	1.506 (2)	C23—C28	1.433 (2)
C2—C3	1.397 (3)	C24—C25	1.439 (2)
C2—C7	1.406 (3)	C25—H25	0.9500
C3—H3	0.9500	C25—C26	1.356 (3)
C3—C4	1.388 (3)	C26—H26	0.9500
C4—H4	0.9500	C26—C27	1.429 (3)
C4—C5	1.391 (3)	C27—C28	1.414 (3)
C5—C6	1.432 (3)	C27—C32	1.402 (3)
C5—C14	1.436 (3)	C28—C29	1.421 (3)
C6—C7	1.430 (3)	C29—C30	1.403 (3)
C6—C11	1.423 (3)	C29—C33	1.440 (3)
C7—C8	1.452 (3)	C30—H30	0.9500
C8—H8	0.9500	C30—C31	1.381 (3)
C8—C9	1.348 (3)	C31—H31	0.9500
C9—H9	0.9500	C31—C32	1.385 (3)
C9—C10	1.440 (3)	C32—H32	0.9500
C10—C11	1.416 (3)	C33—H33	0.9500
C10—C17	1.401 (3)	C33—C34	1.333 (3)
C11—C12	1.427 (3)	C34—H34	0.9500
C12—C13	1.429 (3)	C35—H35A	0.9800
C12—C15	1.402 (3)	C35—H35B	0.9800
C13—H13	0.9500	C35—H35C	0.9800
C13—C14	1.345 (3)	C36—H36A	0.9800
C14—H14	0.9500	C36—H36B	0.9800
C15—H15	0.9500	C36—H36C	0.9800
C15—C16	1.381 (3)		
			
O1—Cu1—Cu1^i^	82.80 (4)	C15—C16—C17	120.4 (2)
O1—Cu1—O2^i^	169.36 (5)	C17—C16—H16	119.8
O1—Cu1—O3	90.10 (6)	C10—C17—H17	119.6
O1—Cu1—O4^i^	90.57 (6)	C16—C17—C10	120.8 (2)
O1—Cu1—O5	93.90 (5)	C16—C17—H17	119.6
O2^i^—Cu1—Cu1^i^	86.57 (4)	O3—C18—C19	118.75 (15)
O2^i^—Cu1—O5	96.67 (5)	O4—C18—O3	124.48 (16)
O3—Cu1—Cu1^i^	85.59 (4)	O4—C18—C19	116.65 (15)
O3—Cu1—O2^i^	89.71 (6)	C20—C19—C18	116.52 (15)
O3—Cu1—O4^i^	169.21 (5)	C20—C19—C24	119.37 (16)
O3—Cu1—O5	97.61 (5)	C24—C19—C18	123.93 (16)
O4^i^—Cu1—Cu1^i^	83.81 (4)	C19—C20—H20	119.2
O4^i^—Cu1—O2^i^	87.65 (6)	C21—C20—C19	121.65 (17)
O4^i^—Cu1—O5	93.09 (5)	C21—C20—H20	119.2
O5—Cu1—Cu1^i^	175.43 (4)	C20—C21—H21	119.6
O5—S3—C35	104.69 (11)	C20—C21—C22	120.87 (17)
O5—S3—C36	104.05 (9)	C22—C21—H21	119.6
C35—S3—C36	98.32 (12)	C21—C22—C23	118.55 (17)
C1—O1—Cu1	125.53 (12)	C21—C22—C34	122.07 (17)
C1—O2—Cu1^i^	119.65 (12)	C23—C22—C34	119.38 (17)
C18—O3—Cu1	122.16 (11)	C22—C23—C24	120.75 (16)
C18—O4—Cu1^i^	123.81 (12)	C22—C23—C28	118.73 (16)
S3—O5—Cu1	121.12 (7)	C24—C23—C28	120.49 (16)
O1—C1—O2	125.44 (16)	C19—C24—C23	118.40 (16)
O1—C1—C2	115.73 (15)	C19—C24—C25	123.84 (16)
O2—C1—C2	118.82 (16)	C23—C24—C25	117.75 (16)
C3—C2—C1	116.26 (16)	C24—C25—H25	119.4
C3—C2—C7	119.57 (17)	C26—C25—C24	121.27 (17)
C7—C2—C1	124.17 (16)	C26—C25—H25	119.4
C2—C3—H3	119.0	C25—C26—H26	119.0
C4—C3—C2	121.93 (18)	C25—C26—C27	121.94 (17)
C4—C3—H3	119.0	C27—C26—H26	119.0
C3—C4—H4	119.9	C28—C27—C26	118.59 (16)
C3—C4—C5	120.23 (17)	C32—C27—C26	122.41 (17)
C5—C4—H4	119.9	C32—C27—C28	119.00 (18)
C4—C5—C6	119.22 (17)	C27—C28—C23	119.75 (17)
C4—C5—C14	121.52 (17)	C27—C28—C29	119.92 (17)
C6—C5—C14	119.22 (18)	C29—C28—C23	120.33 (17)
C7—C6—C5	119.90 (17)	C28—C29—C33	118.50 (17)
C11—C6—C5	119.03 (17)	C30—C29—C28	118.98 (18)
C11—C6—C7	121.07 (17)	C30—C29—C33	122.51 (18)
C2—C7—C6	119.02 (17)	C29—C30—H30	119.7
C2—C7—C8	123.80 (17)	C31—C30—C29	120.68 (19)
C6—C7—C8	117.12 (17)	C31—C30—H30	119.7
C7—C8—H8	119.3	C30—C31—H31	119.7
C9—C8—C7	121.48 (18)	C30—C31—C32	120.63 (18)
C9—C8—H8	119.3	C32—C31—H31	119.7
C8—C9—H9	119.0	C27—C32—H32	119.6
C8—C9—C10	122.02 (19)	C31—C32—C27	120.79 (19)
C10—C9—H9	119.0	C31—C32—H32	119.6
C11—C10—C9	118.25 (18)	C29—C33—H33	119.3
C17—C10—C9	122.62 (19)	C34—C33—C29	121.45 (18)
C17—C10—C11	119.11 (19)	C34—C33—H33	119.3
C6—C11—C12	120.06 (18)	C22—C34—H34	119.2
C10—C11—C6	120.03 (17)	C33—C34—C22	121.56 (18)
C10—C11—C12	119.91 (18)	C33—C34—H34	119.2
C11—C12—C13	119.04 (18)	S3—C35—H35A	109.5
C15—C12—C11	118.54 (19)	S3—C35—H35B	109.5
C15—C12—C13	122.42 (18)	S3—C35—H35C	109.5
C12—C13—H13	119.3	H35A—C35—H35B	109.5
C14—C13—C12	121.39 (18)	H35A—C35—H35C	109.5
C14—C13—H13	119.3	H35B—C35—H35C	109.5
C5—C14—H14	119.4	S3—C36—H36A	109.5
C13—C14—C5	121.22 (19)	S3—C36—H36B	109.5
C13—C14—H14	119.4	S3—C36—H36C	109.5
C12—C15—H15	119.4	H36A—C36—H36B	109.5
C16—C15—C12	121.21 (19)	H36A—C36—H36C	109.5
C16—C15—H15	119.4	H36B—C36—H36C	109.5
C15—C16—H16	119.8		
			
Cu1—O1—C1—O2	0.3 (3)	C13—C12—C15—C16	177.1 (2)
Cu1—O1—C1—C2	−178.52 (11)	C14—C5—C6—C7	−179.52 (16)
Cu1^i^—O2—C1—O1	0.5 (3)	C14—C5—C6—C11	0.3 (3)
Cu1^i^—O2—C1—C2	179.35 (12)	C15—C12—C13—C14	−179.7 (2)
Cu1—O3—C18—O4	1.9 (2)	C15—C16—C17—C10	0.5 (4)
Cu1—O3—C18—C19	−173.80 (11)	C17—C10—C11—C6	−179.6 (2)
Cu1^i^—O4—C18—O3	−4.8 (3)	C17—C10—C11—C12	0.3 (3)
Cu1^i^—O4—C18—C19	171.03 (11)	C18—C19—C20—C21	−170.08 (16)
O1—C1—C2—C3	27.4 (2)	C18—C19—C24—C23	168.60 (15)
O1—C1—C2—C7	−152.14 (18)	C18—C19—C24—C25	−12.7 (3)
O2—C1—C2—C3	−151.58 (17)	C19—C20—C21—C22	0.6 (3)
O2—C1—C2—C7	28.9 (3)	C19—C24—C25—C26	179.09 (16)
O3—C18—C19—C20	155.26 (16)	C20—C19—C24—C23	−6.3 (2)
O3—C18—C19—C24	−19.7 (2)	C20—C19—C24—C25	172.45 (16)
O4—C18—C19—C20	−20.8 (2)	C20—C21—C22—C23	−4.9 (3)
O4—C18—C19—C24	164.21 (16)	C20—C21—C22—C34	174.44 (17)
C1—C2—C3—C4	179.28 (16)	C21—C22—C23—C24	3.6 (2)
C1—C2—C7—C6	177.40 (16)	C21—C22—C23—C28	−177.99 (16)
C1—C2—C7—C8	0.3 (3)	C21—C22—C34—C33	179.60 (18)
C2—C3—C4—C5	3.1 (3)	C22—C23—C24—C19	1.9 (2)
C2—C7—C8—C9	177.8 (2)	C22—C23—C24—C25	−176.86 (15)
C3—C2—C7—C6	−2.1 (3)	C22—C23—C28—C27	178.38 (15)
C3—C2—C7—C8	−179.20 (18)	C22—C23—C28—C29	−2.4 (2)
C3—C4—C5—C6	−1.5 (3)	C23—C22—C34—C33	−1.0 (3)
C3—C4—C5—C14	176.21 (17)	C23—C24—C25—C26	−2.2 (2)
C4—C5—C6—C7	−1.7 (3)	C23—C28—C29—C30	−179.02 (17)
C4—C5—C6—C11	178.10 (17)	C23—C28—C29—C33	0.6 (3)
C4—C5—C14—C13	−176.44 (19)	C24—C19—C20—C21	5.2 (3)
C5—C6—C7—C2	3.5 (3)	C24—C23—C28—C27	−3.2 (2)
C5—C6—C7—C8	−179.18 (17)	C24—C23—C28—C29	175.97 (16)
C5—C6—C11—C10	178.02 (18)	C24—C25—C26—C27	−2.0 (3)
C5—C6—C11—C12	−1.9 (3)	C25—C26—C27—C28	3.7 (3)
C6—C5—C14—C13	1.3 (3)	C25—C26—C27—C32	−176.59 (17)
C6—C7—C8—C9	0.6 (3)	C26—C27—C28—C23	−1.0 (2)
C6—C11—C12—C13	2.0 (3)	C26—C27—C28—C29	179.83 (16)
C6—C11—C12—C15	−178.66 (18)	C26—C27—C32—C31	179.91 (18)
C7—C2—C3—C4	−1.2 (3)	C27—C28—C29—C30	0.2 (3)
C7—C6—C11—C10	−2.2 (3)	C27—C28—C29—C33	179.83 (16)
C7—C6—C11—C12	177.90 (17)	C28—C23—C24—C19	−176.43 (15)
C7—C8—C9—C10	−1.1 (3)	C28—C23—C24—C25	4.8 (2)
C8—C9—C10—C11	0.0 (3)	C28—C27—C32—C31	−0.3 (3)
C8—C9—C10—C17	−178.7 (2)	C28—C29—C30—C31	−0.2 (3)
C9—C10—C11—C6	1.7 (3)	C28—C29—C33—C34	1.0 (3)
C9—C10—C11—C12	−178.40 (19)	C29—C30—C31—C32	−0.1 (3)
C9—C10—C17—C16	177.4 (2)	C29—C33—C34—C22	−0.8 (3)
C10—C11—C12—C13	−177.94 (19)	C30—C29—C33—C34	−179.34 (19)
C10—C11—C12—C15	1.4 (3)	C30—C31—C32—C27	0.4 (3)
C11—C6—C7—C2	−176.30 (17)	C32—C27—C28—C23	179.27 (16)
C11—C6—C7—C8	1.0 (3)	C32—C27—C28—C29	0.1 (3)
C11—C10—C17—C16	−1.3 (4)	C33—C29—C30—C31	−179.79 (19)
C11—C12—C13—C14	−0.4 (3)	C34—C22—C23—C24	−175.77 (16)
C11—C12—C15—C16	−2.3 (3)	C34—C22—C23—C28	2.6 (2)
C12—C13—C14—C5	−1.3 (3)	C35—S3—O5—Cu1	84.29 (13)
C12—C15—C16—C17	1.3 (4)	C36—S3—O5—Cu1	−173.01 (10)

Symmetry code: (i) −*x*+2, −*y*+1, −*z*+1.

### Bis(dimethylformamide)tetrakis(µ-pyrene-1-carboxylato)dicopper(Cu—Cu) (2) Crystal data

**Table:**

[Cu_2_(C_17_H_9_O_2_)_4_(C_3_H_7_NO)_2_]	*F*(000) = 1292
*M**_r_* = 1254.24	*D*_x_ = 1.448 Mg m^−^^3^
Monoclinic, *P*2_1_/*n*	Cu *K*α radiation, λ = 1.54184 Å
*a* = 10.54266 (13) Å	Cell parameters from 4485 reflections
*b* = 21.8888 (2) Å	θ = 0.9–0.9°
*c* = 12.66517 (15) Å	µ = 1.46 mm^−^^1^
β = 100.1160 (11)°	*T* = 300 K
*V* = 2877.25 (6) Å^3^	Block, green
*Z* = 2	0.12 × 0.08 × 0.04 mm

### Bis(dimethylformamide)tetrakis(µ-pyrene-1-carboxylato)dicopper(Cu—Cu) (2) Data collection

**Table:**

Rigaku SuperNova Hypix6000 diffractometer	5246 independent reflections
Radiation source: microsource	4485 reflections with *I* > 2σ(*I*)
Detector resolution: 10.0000 pixels mm^-1^	*R*_int_ = 0.023
ω scans	θ_max_ = 68.4°, θ_min_ = 4.0°
Absorption correction: multi-scan (Blessing, 1995)	*h* = −12→10
*T*_min_ = 0.844, *T*_max_ = 0.944	*k* = −26→26
15433 measured reflections	*l* = −13→15

### Bis(dimethylformamide)tetrakis(µ-pyrene-1-carboxylato)dicopper(Cu—Cu) (2) Refinement

**Table:**

Refinement on *F*^2^	1 restraint
Least-squares matrix: full	Hydrogen site location: inferred from neighbouring sites
*R*[*F*^2^ > 2σ(*F*^2^)] = 0.031	H-atom parameters constrained
*wR*(*F*^2^) = 0.091	*w* = 1/[σ^2^(*F*_o_^2^) + (0.0491*P*)^2^ + 0.4863*P*] where *P* = (*F*_o_^2^ + 2*F*_c_^2^)/3
*S* = 1.07	(Δ/σ)_max_ = 0.001
5246 reflections	Δρ_max_ = 0.20 e Å^−^^3^
447 parameters	Δρ_min_ = −0.28 e Å^−^^3^

### Bis(dimethylformamide)tetrakis(µ-pyrene-1-carboxylato)dicopper(Cu—Cu) (2) Special details

**Table:**

Geometry. All esds (except the esd in the dihedral angle between two l.s. planes) are estimated using the full covariance matrix. The cell esds are taken into account individually in the estimation of esds in distances, angles and torsion angles; correlations between esds in cell parameters are only used when they are defined by crystal symmetry. An approximate (isotropic) treatment of cell esds is used for estimating esds involving l.s. planes.

### Bis(dimethylformamide)tetrakis(µ-pyrene-1-carboxylato)dicopper(Cu—Cu) (2) Fractional atomic coordinates and isotropic or equivalent isotropic displacement parameters (Å^2^)

**Table:**

	*x*	*y*	*z*	*U*_iso_*/*U*_eq_	Occ. (<1)
Cu1	0.10029 (2)	0.46339 (2)	0.01883 (2)	0.03473 (9)	
O1	−0.04514 (13)	0.55286 (6)	−0.15584 (11)	0.0493 (3)	
O2	0.12690 (14)	0.49169 (7)	−0.12241 (11)	0.0535 (4)	
O3	0.03477 (13)	0.59478 (6)	0.04766 (13)	0.0547 (4)	
O4	0.20382 (12)	0.53317 (6)	0.08069 (12)	0.0503 (3)	
O5	0.24588 (12)	0.39180 (6)	0.04172 (11)	0.0495 (3)	
N1	0.2843 (2)	0.29133 (9)	0.0207 (2)	0.0861 (7)	
C1	0.05677 (18)	0.53058 (8)	−0.17817 (15)	0.0413 (4)	
C2	0.09464 (19)	0.55034 (9)	−0.28124 (15)	0.0434 (4)	
C3	−0.0038 (2)	0.55820 (11)	−0.36868 (17)	0.0550 (5)	
H3	−0.089016	0.555364	−0.358896	0.066*	
C4	0.0220 (2)	0.57005 (12)	−0.46922 (17)	0.0609 (6)	
H4	−0.045814	0.575850	−0.526110	0.073*	
C5	0.1484 (2)	0.57351 (10)	−0.48705 (16)	0.0516 (5)	
C6	0.25011 (19)	0.56952 (8)	−0.39786 (15)	0.0425 (4)	
C7	0.22369 (18)	0.55878 (8)	−0.29303 (14)	0.0406 (4)	
C8	0.3304 (2)	0.55944 (10)	−0.20505 (16)	0.0492 (5)	
H8	0.314692	0.554463	−0.135557	0.059*	
C9	0.4526 (2)	0.56709 (10)	−0.22076 (18)	0.0559 (5)	
H9	0.519035	0.567719	−0.161763	0.067*	
C10	0.4827 (2)	0.57427 (10)	−0.32593 (18)	0.0541 (5)	
C11	0.3803 (2)	0.57542 (9)	−0.41406 (16)	0.0477 (5)	
C12	0.4058 (2)	0.58161 (11)	−0.51984 (18)	0.0590 (6)	
C13	0.5344 (3)	0.58709 (14)	−0.5332 (2)	0.0805 (8)	
H13	0.553111	0.591080	−0.601975	0.097*	
C14	0.6329 (3)	0.58669 (16)	−0.4474 (3)	0.0885 (9)	
H14	0.717268	0.590893	−0.458614	0.106*	
C15	0.6092 (2)	0.58014 (13)	−0.3441 (2)	0.0735 (7)	
H15	0.677486	0.579644	−0.286562	0.088*	
C16	0.3008 (3)	0.58189 (13)	−0.60718 (19)	0.0714 (7)	
H16	0.317731	0.583965	−0.676722	0.086*	
C17	0.1801 (3)	0.57930 (12)	−0.59274 (17)	0.0649 (6)	
H17	0.114073	0.581250	−0.651932	0.078*	
C18	0.15237 (17)	0.58494 (8)	0.07943 (14)	0.0390 (4)	
C19	0.23279 (18)	0.63965 (8)	0.11993 (14)	0.0409 (4)	
C20	0.1663 (2)	0.69272 (10)	0.1372 (2)	0.0586 (5)	
H20	0.077020	0.693114	0.118151	0.070*	
C21	0.2276 (2)	0.74439 (10)	0.1813 (2)	0.0676 (6)	
H21	0.179776	0.779236	0.189580	0.081*	
C22	0.3608 (2)	0.74503 (10)	0.21386 (19)	0.0568 (5)	
C23	0.43190 (18)	0.69221 (8)	0.19576 (16)	0.0446 (4)	
C24	0.36931 (17)	0.63922 (8)	0.14546 (14)	0.0386 (4)	
C25	0.44932 (18)	0.58915 (9)	0.12436 (16)	0.0463 (4)	
H25	0.411076	0.554935	0.088547	0.056*	
C26	0.5787 (2)	0.59043 (10)	0.15518 (18)	0.0529 (5)	
H26	0.627295	0.557535	0.138365	0.064*	
C27	0.6423 (2)	0.64062 (10)	0.2125 (2)	0.0576 (5)	
C28	0.5683 (2)	0.69203 (9)	0.23145 (19)	0.0545 (5)	
C30	0.5536 (3)	0.79524 (12)	0.3041 (3)	0.0912 (10)	
H30	0.592741	0.828210	0.343260	0.109*	0.65 (3)
H30A	0.593911	0.829460	0.338355	0.109*	0.35 (3)
C31	0.4277 (3)	0.79689 (11)	0.2668 (3)	0.0789 (8)	
H31	0.381905	0.832360	0.275001	0.095*	
C35	0.2118 (2)	0.34049 (11)	0.0132 (2)	0.0745 (7)	
H35	0.125485	0.335492	−0.017035	0.089*	
C36	0.4184 (3)	0.29451 (15)	0.0634 (3)	0.0917 (9)	
H36A	0.433205	0.280956	0.136652	0.138*	
H36B	0.447514	0.335928	0.060275	0.138*	
H36C	0.465031	0.268801	0.022143	0.138*	
C29A	0.6250 (15)	0.7471 (9)	0.2866 (17)	0.081 (4)	0.65 (3)
C32A	0.7569 (13)	0.7376 (6)	0.3409 (17)	0.094 (4)	0.65 (3)
H32A	0.795517	0.767020	0.388907	0.112*	0.65 (3)
C33A	0.8273 (12)	0.6859 (6)	0.3235 (18)	0.107 (4)	0.65 (3)
H33A	0.914060	0.683192	0.354053	0.128*	0.65 (3)
C34A	0.7689 (13)	0.6389 (5)	0.2613 (15)	0.077 (4)	0.65 (3)
H34A	0.817416	0.604509	0.251930	0.092*	0.65 (3)
C37A	0.225 (3)	0.2297 (6)	0.014 (3)	0.119 (6)	0.45 (5)
H37A	0.133542	0.233293	−0.005127	0.179*	0.45 (5)
H37B	0.246498	0.209958	0.082987	0.179*	0.45 (5)
H37C	0.258081	0.205900	−0.038534	0.179*	0.45 (5)
C29B	0.636 (3)	0.7361 (17)	0.292 (3)	0.072 (5)	0.35 (3)
C32B	0.771 (2)	0.7459 (12)	0.294 (2)	0.083 (5)	0.35 (3)
H32B	0.808613	0.784346	0.306880	0.099*	0.35 (3)
C33B	0.8402 (19)	0.6974 (11)	0.278 (2)	0.083 (5)	0.35 (3)
H33B	0.929280	0.699307	0.298159	0.099*	0.35 (3)
C34B	0.786 (2)	0.6436 (12)	0.233 (2)	0.071 (5)	0.35 (3)
H34B	0.835839	0.611319	0.215515	0.085*	0.35 (3)
C37B	0.2384 (17)	0.2354 (9)	−0.043 (3)	0.138 (7)	0.55 (5)
H37D	0.208109	0.205937	0.003176	0.208*	0.55 (5)
H37E	0.308233	0.218162	−0.072421	0.208*	0.55 (5)
H37F	0.169484	0.246163	−0.099867	0.208*	0.55 (5)

### Bis(dimethylformamide)tetrakis(µ-pyrene-1-carboxylato)dicopper(Cu—Cu) (2) Atomic displacement parameters (Å^2^)

**Table:**

	*U*^11^	*U*^22^	*U*^33^	*U*^12^	*U*^13^	*U*^23^
Cu1	0.03029 (14)	0.02935 (14)	0.04509 (16)	0.00122 (10)	0.00818 (10)	0.00401 (10)
O1	0.0472 (8)	0.0509 (8)	0.0537 (8)	0.0076 (6)	0.0190 (6)	0.0130 (6)
O2	0.0561 (8)	0.0551 (8)	0.0540 (8)	0.0164 (7)	0.0231 (7)	0.0145 (7)
O3	0.0357 (7)	0.0412 (7)	0.0818 (10)	0.0001 (6)	−0.0042 (7)	−0.0097 (7)
O4	0.0357 (7)	0.0342 (7)	0.0776 (9)	−0.0038 (5)	0.0003 (6)	0.0007 (6)
O5	0.0388 (7)	0.0394 (7)	0.0692 (9)	0.0071 (6)	0.0068 (6)	0.0055 (6)
N1	0.0663 (14)	0.0417 (11)	0.146 (2)	0.0161 (10)	0.0074 (14)	0.0052 (12)
C1	0.0434 (10)	0.0361 (9)	0.0454 (10)	−0.0060 (8)	0.0105 (8)	−0.0010 (7)
C2	0.0456 (10)	0.0430 (10)	0.0425 (9)	−0.0020 (8)	0.0104 (8)	0.0025 (8)
C3	0.0405 (11)	0.0712 (14)	0.0531 (11)	−0.0039 (10)	0.0073 (9)	0.0057 (10)
C4	0.0482 (12)	0.0840 (16)	0.0469 (11)	−0.0021 (11)	−0.0019 (9)	0.0100 (11)
C5	0.0543 (12)	0.0574 (12)	0.0426 (10)	−0.0041 (10)	0.0072 (9)	0.0073 (9)
C6	0.0456 (10)	0.0383 (9)	0.0449 (10)	−0.0026 (8)	0.0111 (8)	0.0033 (7)
C7	0.0428 (10)	0.0369 (9)	0.0423 (9)	−0.0026 (8)	0.0084 (8)	0.0023 (7)
C8	0.0515 (12)	0.0514 (11)	0.0435 (10)	−0.0046 (9)	0.0051 (8)	0.0039 (8)
C9	0.0468 (12)	0.0589 (13)	0.0579 (12)	−0.0059 (10)	−0.0023 (9)	0.0066 (10)
C10	0.0446 (11)	0.0504 (12)	0.0681 (13)	−0.0026 (9)	0.0120 (10)	0.0083 (10)
C11	0.0500 (11)	0.0398 (10)	0.0562 (11)	−0.0004 (8)	0.0173 (9)	0.0073 (8)
C12	0.0624 (14)	0.0587 (13)	0.0616 (13)	0.0021 (11)	0.0265 (11)	0.0147 (10)
C13	0.0717 (18)	0.091 (2)	0.0900 (19)	0.0077 (15)	0.0449 (16)	0.0277 (15)
C14	0.0535 (15)	0.108 (2)	0.112 (2)	0.0050 (15)	0.0361 (16)	0.0313 (19)
C15	0.0440 (12)	0.0824 (18)	0.0947 (19)	−0.0009 (12)	0.0138 (12)	0.0180 (14)
C16	0.0892 (19)	0.0800 (17)	0.0493 (13)	−0.0006 (14)	0.0238 (12)	0.0166 (11)
C17	0.0734 (16)	0.0784 (16)	0.0419 (11)	−0.0036 (13)	0.0074 (10)	0.0133 (10)
C18	0.0341 (9)	0.0393 (10)	0.0433 (9)	−0.0030 (7)	0.0058 (7)	0.0026 (7)
C19	0.0398 (10)	0.0353 (9)	0.0468 (9)	−0.0033 (8)	0.0058 (7)	−0.0005 (7)
C20	0.0400 (11)	0.0484 (12)	0.0834 (15)	0.0029 (9)	0.0002 (10)	−0.0094 (11)
C21	0.0535 (13)	0.0413 (11)	0.1047 (18)	0.0072 (10)	0.0052 (12)	−0.0174 (12)
C22	0.0525 (12)	0.0392 (10)	0.0766 (14)	−0.0029 (9)	0.0060 (10)	−0.0102 (10)
C23	0.0421 (10)	0.0358 (9)	0.0555 (11)	−0.0051 (8)	0.0078 (8)	−0.0017 (8)
C24	0.0387 (9)	0.0344 (9)	0.0433 (9)	−0.0040 (7)	0.0086 (7)	−0.0004 (7)
C25	0.0418 (10)	0.0394 (10)	0.0590 (11)	−0.0062 (8)	0.0127 (9)	−0.0089 (8)
C26	0.0429 (11)	0.0416 (10)	0.0765 (14)	−0.0016 (9)	0.0163 (10)	−0.0092 (9)
C27	0.0403 (11)	0.0464 (11)	0.0840 (15)	−0.0054 (9)	0.0052 (10)	−0.0069 (11)
C28	0.0440 (11)	0.0393 (11)	0.0772 (14)	−0.0071 (9)	0.0021 (10)	−0.0063 (10)
C30	0.0704 (18)	0.0472 (14)	0.146 (3)	−0.0085 (13)	−0.0075 (17)	−0.0371 (16)
C31	0.0702 (17)	0.0391 (12)	0.122 (2)	−0.0008 (11)	0.0027 (15)	−0.0243 (13)
C35	0.0462 (12)	0.0460 (13)	0.125 (2)	0.0083 (10)	−0.0034 (13)	0.0042 (14)
C36	0.0719 (18)	0.083 (2)	0.118 (2)	0.0415 (16)	0.0091 (16)	0.0200 (17)
C29A	0.048 (5)	0.039 (7)	0.143 (7)	−0.010 (6)	−0.018 (4)	−0.025 (5)
C32A	0.056 (5)	0.065 (4)	0.146 (10)	−0.013 (3)	−0.021 (6)	−0.027 (6)
C33A	0.044 (4)	0.073 (4)	0.186 (12)	−0.007 (3)	−0.026 (5)	−0.008 (7)
C34A	0.042 (4)	0.049 (3)	0.139 (10)	−0.002 (3)	0.012 (4)	−0.009 (4)
C37A	0.137 (11)	0.038 (4)	0.174 (15)	0.001 (5)	0.006 (11)	0.011 (6)
C29B	0.059 (8)	0.036 (10)	0.118 (11)	0.018 (6)	0.005 (7)	−0.011 (6)
C32B	0.050 (5)	0.073 (9)	0.122 (14)	−0.027 (5)	0.007 (8)	−0.034 (9)
C33B	0.043 (5)	0.079 (11)	0.124 (12)	−0.022 (6)	0.009 (7)	−0.040 (9)
C34B	0.025 (5)	0.088 (11)	0.099 (10)	−0.011 (5)	0.013 (5)	−0.040 (8)
C37B	0.133 (8)	0.050 (5)	0.239 (19)	0.009 (5)	0.051 (12)	−0.011 (10)

### Bis(dimethylformamide)tetrakis(µ-pyrene-1-carboxylato)dicopper(Cu—Cu) (2) Geometric parameters (Å, º)

**Table:**

Cu1—Cu1^i^	2.6295 (5)	C20—H20	0.9300
Cu1—O1^i^	1.9570 (13)	C20—C21	1.372 (3)
Cu1—O2	1.9593 (13)	C21—H21	0.9300
Cu1—O3^i^	1.9841 (13)	C21—C22	1.393 (3)
Cu1—O4	1.9590 (13)	C22—C23	1.418 (3)
Cu1—O5	2.1769 (13)	C22—C31	1.439 (3)
O1—C1	1.257 (2)	C23—C24	1.428 (2)
O2—C1	1.260 (2)	C23—C28	1.430 (3)
O3—C18	1.253 (2)	C24—C25	1.436 (3)
O4—C18	1.255 (2)	C25—H25	0.9300
O5—C35	1.215 (3)	C25—C26	1.351 (3)
N1—C35	1.313 (3)	C26—H26	0.9300
N1—C36	1.425 (4)	C26—C27	1.419 (3)
N1—C37A	1.481 (10)	C27—C28	1.414 (3)
N1—C37B	1.498 (11)	C27—C34A	1.369 (14)
C1—C2	1.495 (3)	C27—C34B	1.49 (2)
C2—C3	1.389 (3)	C28—C29A	1.466 (15)
C2—C7	1.407 (3)	C28—C29B	1.35 (3)
C3—H3	0.9300	C30—H30	0.9300
C3—C4	1.373 (3)	C30—H30A	0.9300
C4—H4	0.9300	C30—C31	1.329 (4)
C4—C5	1.392 (3)	C30—C29A	1.34 (2)
C5—C6	1.417 (3)	C30—C29B	1.59 (4)
C5—C17	1.441 (3)	C31—H31	0.9300
C6—C7	1.423 (3)	C35—H35	0.9300
C6—C11	1.429 (3)	C36—H36A	0.9600
C7—C8	1.438 (3)	C36—H36B	0.9600
C8—H8	0.9300	C36—H36C	0.9600
C8—C9	1.349 (3)	C29A—C32A	1.45 (2)
C9—H9	0.9300	C32A—H32A	0.9300
C9—C10	1.431 (3)	C32A—C33A	1.391 (18)
C10—C11	1.410 (3)	C33A—H33A	0.9300
C10—C15	1.398 (3)	C33A—C34A	1.374 (18)
C11—C12	1.418 (3)	C34A—H34A	0.9300
C12—C13	1.401 (3)	C37A—H37A	0.9600
C12—C16	1.421 (4)	C37A—H37B	0.9600
C13—H13	0.9300	C37A—H37C	0.9600
C13—C14	1.365 (4)	C29B—C32B	1.43 (4)
C14—H14	0.9300	C32B—H32B	0.9300
C14—C15	1.382 (4)	C32B—C33B	1.32 (3)
C15—H15	0.9300	C33B—H33B	0.9300
C16—H16	0.9300	C33B—C34B	1.39 (3)
C16—C17	1.318 (4)	C34B—H34B	0.9300
C17—H17	0.9300	C37B—H37D	0.9600
C18—C19	1.504 (2)	C37B—H37E	0.9600
C19—C20	1.394 (3)	C37B—H37F	0.9600
C19—C24	1.419 (3)		
			
O1^i^—Cu1—Cu1^i^	85.23 (4)	C21—C20—H20	118.7
O1^i^—Cu1—O2	168.37 (6)	C20—C21—H21	119.8
O1^i^—Cu1—O3^i^	87.53 (7)	C20—C21—C22	120.5 (2)
O1^i^—Cu1—O4	91.06 (6)	C22—C21—H21	119.8
O1^i^—Cu1—O5	93.64 (5)	C21—C22—C23	118.57 (19)
O2—Cu1—Cu1^i^	83.18 (4)	C21—C22—C31	122.2 (2)
O2—Cu1—O3^i^	91.29 (7)	C23—C22—C31	119.2 (2)
O2—Cu1—O5	97.96 (5)	C22—C23—C24	121.22 (18)
O3^i^—Cu1—Cu1^i^	79.62 (4)	C22—C23—C28	118.82 (18)
O3^i^—Cu1—O5	91.79 (5)	C24—C23—C28	119.94 (17)
O4—Cu1—Cu1^i^	88.35 (4)	C19—C24—C23	117.85 (16)
O4—Cu1—O2	87.68 (7)	C19—C24—C25	124.61 (16)
O4—Cu1—O3^i^	167.96 (5)	C23—C24—C25	117.54 (17)
O4—Cu1—O5	100.23 (5)	C24—C25—H25	119.1
O5—Cu1—Cu1^i^	171.37 (4)	C26—C25—C24	121.75 (18)
C1—O1—Cu1^i^	121.86 (12)	C26—C25—H25	119.1
C1—O2—Cu1	124.16 (12)	C25—C26—H26	119.1
C18—O3—Cu1^i^	128.45 (12)	C25—C26—C27	121.77 (19)
C18—O4—Cu1	119.31 (12)	C27—C26—H26	119.1
C35—O5—Cu1	117.47 (14)	C26—C27—C34B	119.7 (9)
C35—N1—C36	120.9 (2)	C28—C27—C26	118.53 (19)
C35—N1—C37A	120.6 (11)	C28—C27—C34B	120.7 (10)
C35—N1—C37B	120.2 (10)	C34A—C27—C26	123.1 (6)
C36—N1—C37A	116.3 (9)	C34A—C27—C28	117.9 (6)
C36—N1—C37B	116.7 (8)	C23—C28—C29A	116.8 (8)
O1—C1—O2	125.24 (17)	C27—C28—C23	120.21 (18)
O1—C1—C2	117.02 (17)	C27—C28—C29A	122.9 (8)
O2—C1—C2	117.69 (16)	C29B—C28—C23	125.5 (15)
C3—C2—C1	117.09 (17)	C29B—C28—C27	114.1 (16)
C3—C2—C7	120.03 (17)	C31—C30—H30	119.4
C7—C2—C1	122.85 (17)	C31—C30—H30A	119.3
C2—C3—H3	119.3	C31—C30—C29A	121.2 (6)
C4—C3—C2	121.4 (2)	C31—C30—C29B	121.3 (11)
C4—C3—H3	119.3	C29A—C30—H30	119.4
C3—C4—H4	119.6	C29B—C30—H30A	119.3
C3—C4—C5	120.8 (2)	C22—C31—H31	119.3
C5—C4—H4	119.6	C30—C31—C22	121.5 (2)
C4—C5—C6	118.65 (18)	C30—C31—H31	119.3
C4—C5—C17	122.7 (2)	O5—C35—N1	126.8 (2)
C6—C5—C17	118.6 (2)	O5—C35—H35	116.6
C5—C6—C7	120.60 (18)	N1—C35—H35	116.6
C5—C6—C11	119.45 (17)	N1—C36—H36A	109.5
C7—C6—C11	119.95 (18)	N1—C36—H36B	109.5
C2—C7—C6	118.17 (17)	N1—C36—H36C	109.5
C2—C7—C8	123.96 (17)	H36A—C36—H36B	109.5
C6—C7—C8	117.83 (17)	H36A—C36—H36C	109.5
C7—C8—H8	119.2	H36B—C36—H36C	109.5
C9—C8—C7	121.65 (19)	C30—C29A—C28	122.2 (11)
C9—C8—H8	119.2	C30—C29A—C32A	123.7 (12)
C8—C9—H9	119.2	C32A—C29A—C28	112.6 (15)
C8—C9—C10	121.7 (2)	C29A—C32A—H32A	118.8
C10—C9—H9	119.2	C33A—C32A—C29A	122.3 (12)
C11—C10—C9	118.33 (19)	C33A—C32A—H32A	118.8
C15—C10—C9	122.5 (2)	C32A—C33A—H33A	120.0
C15—C10—C11	119.2 (2)	C34A—C33A—C32A	120.1 (10)
C10—C11—C6	120.39 (18)	C34A—C33A—H33A	120.0
C10—C11—C12	120.24 (19)	C27—C34A—C33A	122.8 (10)
C12—C11—C6	119.4 (2)	C27—C34A—H34A	118.6
C11—C12—C16	119.0 (2)	C33A—C34A—H34A	118.6
C13—C12—C11	118.1 (2)	N1—C37A—H37A	109.5
C13—C12—C16	122.9 (2)	N1—C37A—H37B	109.5
C12—C13—H13	119.3	N1—C37A—H37C	109.5
C14—C13—C12	121.3 (2)	H37A—C37A—H37B	109.5
C14—C13—H13	119.3	H37A—C37A—H37C	109.5
C13—C14—H14	119.5	H37B—C37A—H37C	109.5
C13—C14—C15	121.1 (2)	C28—C29B—C30	113 (2)
C15—C14—H14	119.5	C28—C29B—C32B	123 (3)
C10—C15—H15	120.0	C32B—C29B—C30	116 (2)
C14—C15—C10	120.1 (3)	C29B—C32B—H32B	121.8
C14—C15—H15	120.0	C33B—C32B—C29B	116 (2)
C12—C16—H16	119.0	C33B—C32B—H32B	121.8
C17—C16—C12	122.1 (2)	C32B—C33B—H33B	118.4
C17—C16—H16	119.0	C32B—C33B—C34B	123 (2)
C5—C17—H17	119.3	C34B—C33B—H33B	118.4
C16—C17—C5	121.3 (2)	C27—C34B—H34B	121.9
C16—C17—H17	119.3	C33B—C34B—C27	116.1 (18)
O3—C18—O4	124.08 (17)	C33B—C34B—H34B	121.9
O3—C18—C19	116.05 (16)	N1—C37B—H37D	109.5
O4—C18—C19	119.86 (16)	N1—C37B—H37E	109.5
C20—C19—C18	116.64 (17)	N1—C37B—H37F	109.5
C20—C19—C24	119.19 (17)	H37D—C37B—H37E	109.5
C24—C19—C18	124.11 (16)	H37D—C37B—H37F	109.5
C19—C20—H20	118.7	H37E—C37B—H37F	109.5
C21—C20—C19	122.5 (2)		
			
Cu1^i^—O1—C1—O2	7.3 (3)	C19—C20—C21—C22	−1.9 (4)
Cu1^i^—O1—C1—C2	−175.04 (12)	C19—C24—C25—C26	−176.85 (19)
Cu1—O2—C1—O1	−6.1 (3)	C20—C19—C24—C23	4.7 (3)
Cu1—O2—C1—C2	176.21 (13)	C20—C19—C24—C25	−175.3 (2)
Cu1^i^—O3—C18—O4	−5.2 (3)	C20—C21—C22—C23	2.9 (4)
Cu1^i^—O3—C18—C19	176.02 (12)	C20—C21—C22—C31	−175.9 (3)
Cu1—O4—C18—O3	5.4 (3)	C21—C22—C23—C24	−0.1 (3)
Cu1—O4—C18—C19	−175.92 (12)	C21—C22—C23—C28	−178.4 (2)
Cu1—O5—C35—N1	−179.5 (3)	C21—C22—C31—C30	175.1 (3)
O1—C1—C2—C3	−38.1 (3)	C22—C23—C24—C19	−3.7 (3)
O1—C1—C2—C7	144.29 (19)	C22—C23—C24—C25	176.31 (19)
O2—C1—C2—C3	139.8 (2)	C22—C23—C28—C27	−178.6 (2)
O2—C1—C2—C7	−37.9 (3)	C22—C23—C28—C29A	1.0 (11)
O3—C18—C19—C20	11.8 (3)	C22—C23—C28—C29B	7 (2)
O3—C18—C19—C24	−171.15 (17)	C23—C22—C31—C30	−3.6 (5)
O4—C18—C19—C20	−167.00 (19)	C23—C24—C25—C26	3.1 (3)
O4—C18—C19—C24	10.1 (3)	C23—C28—C29A—C30	1 (2)
C1—C2—C3—C4	−173.2 (2)	C23—C28—C29A—C32A	166.7 (14)
C1—C2—C7—C6	171.59 (17)	C23—C28—C29B—C30	−10 (3)
C1—C2—C7—C8	−10.8 (3)	C23—C28—C29B—C32B	−157 (3)
C2—C3—C4—C5	1.2 (4)	C24—C19—C20—C21	−2.1 (3)
C2—C7—C8—C9	179.3 (2)	C24—C23—C28—C27	3.0 (3)
C3—C2—C7—C6	−6.0 (3)	C24—C23—C28—C29A	−177.4 (10)
C3—C2—C7—C8	171.7 (2)	C24—C23—C28—C29B	−171 (2)
C3—C4—C5—C6	−5.1 (4)	C24—C25—C26—C27	1.7 (3)
C3—C4—C5—C17	173.2 (2)	C25—C26—C27—C28	−4.1 (4)
C4—C5—C6—C7	3.5 (3)	C25—C26—C27—C34A	168.0 (9)
C4—C5—C6—C11	−177.5 (2)	C25—C26—C27—C34B	−172.2 (13)
C4—C5—C17—C16	−179.4 (3)	C26—C27—C28—C23	1.6 (3)
C5—C6—C7—C2	2.0 (3)	C26—C27—C28—C29A	−177.9 (11)
C5—C6—C7—C8	−175.78 (18)	C26—C27—C28—C29B	176.7 (18)
C5—C6—C11—C10	177.22 (19)	C26—C27—C34A—C33A	−174.9 (9)
C5—C6—C11—C12	−3.6 (3)	C26—C27—C34B—C33B	172.8 (14)
C6—C5—C17—C16	−1.1 (4)	C27—C28—C29A—C30	−179.8 (12)
C6—C7—C8—C9	−3.0 (3)	C27—C28—C29A—C32A	−14 (2)
C6—C11—C12—C13	−179.8 (2)	C27—C28—C29B—C30	175.6 (15)
C6—C11—C12—C16	0.1 (3)	C27—C28—C29B—C32B	28 (4)
C7—C2—C3—C4	4.5 (3)	C28—C23—C24—C19	174.60 (18)
C7—C6—C11—C10	−3.7 (3)	C28—C23—C24—C25	−5.3 (3)
C7—C6—C11—C12	175.38 (19)	C28—C27—C34A—C33A	−2.8 (14)
C7—C8—C9—C10	−0.7 (3)	C28—C27—C34B—C33B	5 (2)
C8—C9—C10—C11	2.3 (3)	C28—C29A—C32A—C33A	12 (2)
C8—C9—C10—C15	−177.7 (2)	C28—C29B—C32B—C33B	−30 (5)
C9—C10—C11—C6	0.0 (3)	C30—C29A—C32A—C33A	177.8 (14)
C9—C10—C11—C12	−179.1 (2)	C30—C29B—C32B—C33B	−176.5 (19)
C9—C10—C15—C14	179.7 (3)	C31—C22—C23—C24	178.8 (2)
C10—C11—C12—C13	−0.6 (3)	C31—C22—C23—C28	0.4 (3)
C10—C11—C12—C16	179.3 (2)	C31—C30—C29A—C28	−4 (2)
C11—C6—C7—C2	−177.02 (17)	C31—C30—C29A—C32A	−168.4 (17)
C11—C6—C7—C8	5.2 (3)	C31—C30—C29B—C28	6 (3)
C11—C10—C15—C14	−0.3 (4)	C31—C30—C29B—C32B	156 (2)
C11—C12—C13—C14	−0.2 (4)	C36—N1—C35—O5	−1.6 (5)
C11—C12—C16—C17	3.1 (4)	C29A—C30—C31—C22	5.4 (13)
C12—C13—C14—C15	0.8 (5)	C29A—C32A—C33A—C34A	−6 (2)
C12—C16—C17—C5	−2.6 (4)	C32A—C33A—C34A—C27	1.2 (17)
C13—C12—C16—C17	−177.0 (3)	C34A—C27—C28—C23	−170.8 (9)
C13—C14—C15—C10	−0.5 (5)	C34A—C27—C28—C29A	9.7 (15)
C15—C10—C11—C6	180.0 (2)	C37A—N1—C35—O5	161.2 (16)
C15—C10—C11—C12	0.9 (3)	C29B—C30—C31—C22	0.4 (17)
C16—C12—C13—C14	179.9 (3)	C29B—C32B—C33B—C34B	17 (3)
C17—C5—C6—C7	−174.9 (2)	C32B—C33B—C34B—C27	−6 (3)
C17—C5—C6—C11	4.2 (3)	C34B—C27—C28—C23	169.7 (12)
C18—C19—C20—C21	175.1 (2)	C34B—C27—C28—C29B	−15 (2)
C18—C19—C24—C23	−172.23 (17)	C37B—N1—C35—O5	−163.9 (17)
C18—C19—C24—C25	7.7 (3)		

Symmetry code: (i) −*x*, −*y*+1, −*z*.
